# Assessment of mosquito species communities biting humans and their livestock in the forest hills of Karen state, Myanmar: a cross-sectional survey in six villages

**DOI:** 10.1186/s13071-025-07217-9

**Published:** 2025-12-29

**Authors:** Victor Chaumeau, Thithiwarada Kularbkeeree, Naw Gloria, Naw Jaruwan, Sunisa Sawasdichai, Chanapat Pateekhum, Florian Girond, Vincent Herbreteau, François Nosten

**Affiliations:** 1 Shoklo Malaria Research Unit, Mahidol-Oxford Tropical Medicine Research Unit, Faculty of Tropical Medicine, Mahidol University, Mae Ramat, Thailand; 2 Centre for Tropical Medicine and Global Health, Nuffield Department of Medicine, University of Oxford, Oxford, UK; 3 Communicable Disease Control Department, Ministry of Health, Phnom Penh, Cambodia; 4 Institut de Recherche pour le Développement, UMR 228 Espace-Dev, Phnom Penh, Cambodia

**Keywords:** *Culicidae*, Species community, Biodiversity, Southeast Asia, Forest, Generalized linear latent variable model, Remote sensing, Mosquito-borne diseases

## Abstract

**Background:**

Mosquito-borne diseases cause significant burdens in rural areas of Southeast Asia. The lack of data on vector bionomics hinders disease control and elimination. The objectives of this study were to assess the diversity and biting behaviours of mosquito species biting humans and their livestock in the forest hills of Karen state, Myanmar, and to assess the patterns of species co-occurrence and the effects of the environment on vector abundance.

**Methods:**

Mosquitoes were captured over 24-h diel cycles in six villages in September 2019 using the human landing catch and cow-baited trap collection methods. Collected specimens were identified to the species level using dichotomous morphological keys. Environmental data were acquired through remote sensing. The analysis of biting times was performed with circular statistics. Species co-occurrence patterns and the effects of environmental variables on species abundance were assessed with a generalized linear latent variable model.

**Results:**

A total of 36,607 mosquitoes were captured, and 96 species in 16 genera were identified. The most abundant genera were *Anopheles*, *Culex* and *Downsiomyia*. Multiple malaria, arboviruses and lymphatic filariasis vector species were detected, and their biting behaviours were reported. Generalized linear latent variable modelling revealed two clusters of species that were positively correlated with one another. The first cluster included many *Culex* and *Anopheles* species, and *Mansonia annulata*, which breed in shallow, stagnant or slow-moving water, such as marshes, swamps, rice fields and the margins of streams and puddles. These species were negatively associated with elevation, slopes and forests and positively associated with grasslands, shrubs and crop fields. The second cluster included many rainforest mosquitoes of the genera *Armigeres*, *Heizmannia*, *Downsiomyia*, *Anopheles dirus*, *An. jeyporiensis*, *Culex bitaeniorhynchus* and *Aedes pseudoalbopictus*, which breed in tree canopies and in natural surface water and containers such as bamboo stumps, tree holes and rainwater pools. These species were positively associated with elevation, slope, dense forests, surface water and wetlands and negatively associated with crop fields, grasslands and shrubs.

**Conclusions:**

Transmission dynamics are particularly complex in this setting where people are exposed to bites of numerous vector species throughout the diel cycle. Environmental factors shape the assembly of mosquito species communities and largely determine the risk of exposure to vector bites.

**Graphical abstract:**

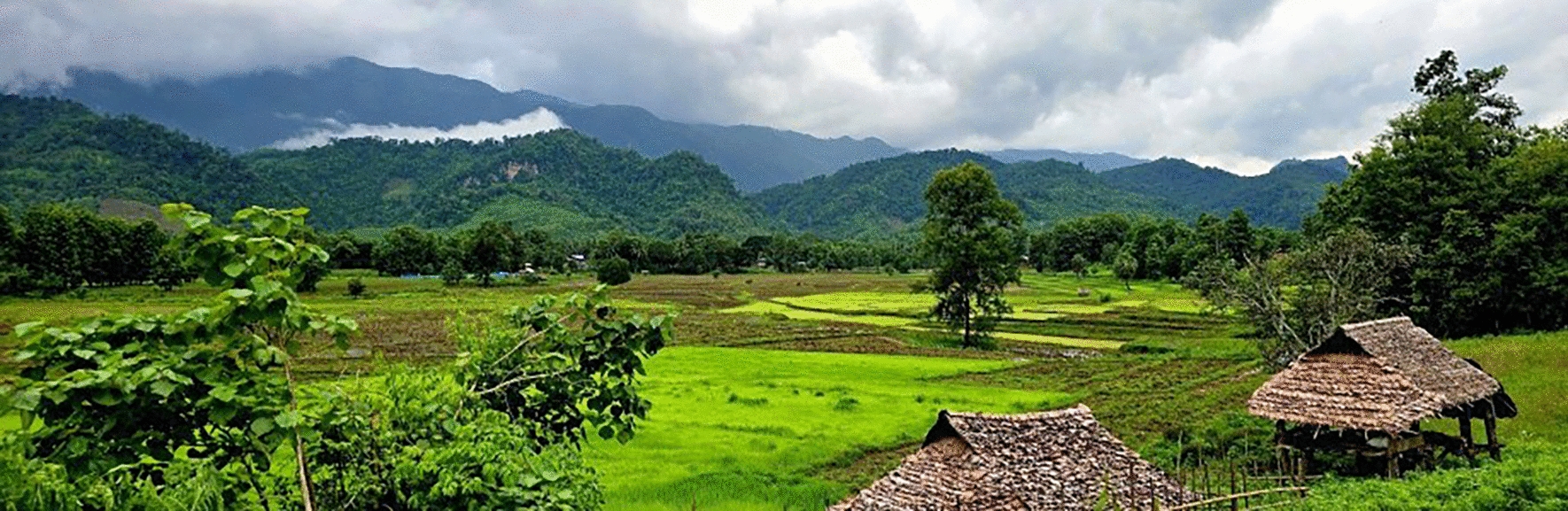

**Supplementary Information:**

The online version contains supplementary material available at 10.1186/s13071-025-07217-9.

## Background

The Thailand-Myanmar border is a rural area of Southeast Asia where people and animals are exposed to bites of vector mosquitoes, causing important burdens of mosquito-borne diseases in the affected communities [1].

Malaria was the leading cause of death in the general population 3 decades ago [2]. Its endemicity has drastically declined in recent years [3], but multi-drug resistant falciparum malaria and relapsing vivax malaria remain important public health concerns [4]. The main local vectors are *Anopheles dirus*, *An. baimai* (Leucosphyrus Group, Dirus Complex), *An. minimus* (Funestus Group, Minimus Complex), *An. maculatus* and *An. sawadwongporni* (Maculatus Group). Several other species play a secondary role in the transmission or are suspected vectors because they transmit malaria elsewhere, but their vector status at the Thailand-Myanmar border has not been characterised [5].

Dengue, chikungunya and Zika viruses are mainly transmitted by *Aedes* mosquitoes [6–8]. The main vectors are *Aedes aegypti* and *Ae. albopictus* in urban and rural areas, respectively [9]. The vector status of other species is not well known. *Downsiomyia niveoides* contributes to sylvatic dengue cycles [10]. *Armigeres subalbatus* and *Anopheles stephensi* can transmit Zika and chikungunya viruses under laboratory conditions, respectively [11, 12].

Japanese encephalitis is a leading cause of severe central nervous system infection in Asia. Most cases are asymptomatic, but approximately 30% of the symptomatic cases are fatal and 50% of the survivors suffer from permanent sequelae [13]. *Culex tritaeniorhynchus*, *Cx. vishnui* and *Cx. gelidus* are the main vectors but Japanese encephalitis viruses can infect many other mosquito species that may also be involved in natural transmission cycles [14].

Focal transmission of lymphatic filariasis occurs in some villages where local ecological factors determine vector occurrence and filariasis type [15]. Nocturnal subperiodic *Wuchereria bancrofti* is transmitted by the rainforest mosquitoes *Downsiomyia harinasutai*, *Aedes annandalei*, *Ae. imitator*, *Ae. desmotes* and *Mansonia dives* [16]. Periodic *W. bancrofti* transmitted by *Culex quinquefasciatus* in urban areas of Myanmar has the potential to spread in cities in Thailand where local *Cx. quinquefasciatus* populations are capable vectors [17]. Nocturnal periodic *Brugia malayi* is transmitted in swamp forests (*Mansonia uniformis* type) and in rice fields (*Anopheles barbirostris* type) by several *Anopheles*, *Culex* and *Mansonia* species [18]. Rare cases of infection with *Brugia pahangi*, *Dirofilaria immitis*, *D. repens* and *Dirofilaria* sp. “*hongkongensis”* have also been reported [19–23]. *Armigeres subalbatus*, *Cx. quinquefasciatus* and *Ma. uniformis* were identified as important natural vectors [24–29].

This study sought to describe the diversity and biting behaviours of mosquito species biting humans and their livestock in villages located in forested areas of Karen (Kayin) state, Myanmar, and to assess the patterns of species co-occurrence and the effects of environmental variables on vector abundance.

## Methods

### Study villages, participants and interventions

The six villages selected for this study were malaria hotspots (defined by a high prevalence of submicroscopic malaria infections) located in forested areas of Karen State, Myanmar (17.90°N, 97.25°E to 17.70°N, 97.60°E). These villages were participating in a malaria elimination programme started in 2014. The study protocol (including details of informed consent, eligibility criteria, laboratory procedures and antimalarial drug regimens) and main findings were reported previously [4, 30]. Briefly, community-wide access to early malaria diagnosis and treatment was provided in all villages, and mass antimalarial drug administration campaigns were conducted in villages with high prevalence of submicroscopic infections. Mosquitoes were captured to assess the entomological indices of transmission. Detection of microfilariae in participants’ blood samples was incidental and motivated additional mass antifilarial drug administration campaigns following the Myanmar National Plan to Eliminate Lymphatic Filariasis [31].

### Population census and passive malaria case detection

At the beginning of the programme, a census was conducted, and the age, sex and household number of all inhabitants were recorded. A malaria post was set up, and villagers were asked to consult the malaria post worker if they had fever within 48 h of symptom onset. All fever cases were tested with malarial rapid diagnostic tests, and malaria cases were treated accordingly. All cases were recorded and reported to the epidemiological surveillance system of the programme, allowing estimation of incidence rates.

### Blood surveys

Cross-sectional blood surveys were conducted in a subset of the adult population between March 2017 and December 2018 to assess the prevalence of submicroscopic *Plasmodium* infections with a highly sensitive RT-qPCR assay detecting parasite RNA (estimated limit of detection: 22 parasites per ml of blood) [32]. A thin smear and a tick drop were also made from capillary blood samples. The Giemsa-stained slides were initially examined for malaria to estimate the proportion of submicroscopic infections among participants with positive *Plasmodium* PCR results. The detection of microfilariae was incidental, and filariae species were identified using standard criteria [33].

### Entomological surveys

Entomological surveys were conducted in every village between September 13, 2019, and September 26, 2019. Mosquitoes were captured during 24-h diel cycles for 5 consecutive days in each village. Collectors were villagers aged at least 18 years old. Four teams rostered over six 6-h shifts to cover the entire diel. In each village, five houses were randomly selected for mosquito sampling using the human landing catch collection method. In each house, one mosquito collector sat indoors (in the living room) and another sat outdoors (10–20 m from the house), yielding a total of 50 person-days of collection per survey (25 person-days indoors and 25 person-days outdoors). Hence, indoor and outdoor collection sites were representative of traditional housing in this region and of the peri-domestic environment of the villages. Collectors were asked to catch every mosquito landing on their uncovered legs for 50 min per hour and allowed to rest for 10 min per hour. A cow-baited trap was also set up in each village, yielding an additional 5 cow-nights of collection per survey. One cow was isolated and placed approximately 100 m from the herd, and a 1-m-wide mosquito net was fenced around the animal 30 cm above the ground level. One collector was asked to capture mosquitoes resting on the net for 50 min per hour and allowed to rest for 10 min. Mosquitoes were collected individually into 5-ml plastic tubes and shipped to the Shoklo Malaria Research Unit headquarters at the end of the survey. Upon reception at the laboratory, mosquitoes were killed by freezing them at −20 °C. Then, dead mosquitoes were transferred into 1.5-ml plastic tubes filled with silica gel and cotton and stored at −20 °C until they were identified with dichotomic morphological identification keys [34–39]. All specimens were identified by three laboratory technicians, and the consensus result was reported.

### Environmental data

Environmental data included in this analysis were: length of rivers and streams, elevation, slope, deforestation patterns, land use, land cover, normalized difference vegetation index (NDVI), modified normalized difference water index (MNDWI) and Gao’s normalized difference water index (NDWIGAO) [40], daily rainfall, mean air and mean dewpoint temperatures. They were obtained using remote sensing methods as described. For each variable, the data were extracted within a 1-km radius from each village.

The length of rivers and streams was obtained from OpenStreetMap data using the *osmdata* R package [41]. Elevation, slope and deforestation patterns were obtained using the *terra* R package [42] with data from NASA’s Earth Science Data Systems programme and the Hansen Global Forest Change 2000–2023 dataset provided by the Department of Geographical Sciences of the University of Maryland. A land use-land cover data map was made by analysing Sentinel-2 satellite images from 2019 and 2020 using object-based image analysis method with eCognition software, version 9.0.3. These images were provided by the Copernicus Programme from the European Space Agency. They had a high spatial and temporal resolution (10–60 m, and 5 days respectively) allowing selection of cloud-free scenes. The map was produced over the entire Karen State and validated with field observation at 300 random locations and 300 points photo-interpreted from Google Earth [43]. NDVI, MNDWI and NDWIGAO indices were calculated using Sentinel-2 satellite images acquired weekly during the month before entomological surveys. Daily rainfall data and the mean air and dewpoint temperatures were collected using a combination of R and Google Earth Engine with the *rgee* R package [44].

### Data analysis

The analysis was carried out with R version 4.4 [45]. Species diversity indices were estimated using the *iNEXT* package as described previously [46]. Analysis of mosquito biting times was carried out using the *circular* package [47]. Discrete mosquito biting times aggregated over 1-h time slots at collection were transformed to a pseudocontinuous variable by adding a random component over the [0,1] interval using a random number generator. Under the circular probability model, the likelihood function for pseudocontinuous biting times was a von Mises distribution parameterized by its mean direction *μ* and concentration *κ*. Values of *κ* closer to 1 indicate greater concentration of events, whereas values of *κ* closer to 0 indicate sparser events. When *κ* = 0, events are uniformly distributed and the mean direction is not defined, meaning that the mosquito biting rate is constant throughout the 24-h diel cycle. Comparison of biting activity across villages and collection methods did not show significant differences, so biting events were pooled in the analysis. Analysis of mosquito species community assembly was performed using the *gllvm* package [48]. A generalized linear latent variable model was fitted to a multivariate abundance dataset represented as a count matrix with *n* rows corresponding to collection sites and *m* columns corresponding to species. In the model, the mean count *μ*_*i,j*_ for the *j*th species at the *i*th site is regressed against a set of *k* environmental variables recorded at each site *x*_*i*_ = (*x*_*i1*_, …, *x*_*ik*_)^⊤^ and a vector of *d* <  < *m* latent variables *u*_*i*_ = (*u*_*i1*_, …, *u*_*id*_)^⊤^:

$$g\left( {\mu_{i,j} } \right) \, = \, \alpha_{i} + \, \beta_{0j} + \, x_{i}^{ \top } \beta_{j} + \, u_{i}^{ \top } \gamma_{j} ,$$

where *α*_*i*_ are random site effects such as *α*_*i*_ ∼ N(0, *σ*^*2*^), *β*_*0j*_ are species-specific intercepts, and *β*_*j*_ and *γ*_*j*_ are vectors of species-specific coefficients related to the covariates and latent variables, respectively. To account for overdispersion, the likelihood for the mean mosquito count was a negative binomial distribution parameterized by its mean *μ*_*i,j*_ and dispersion *Φ* = *(φ*_*1*_, …, *φ*_*m*_*)*^*⊤*^. Environmental variables included in the model were elevation, slope, number of households, length of rivers and streams, deforestation index, and land use and land cover classes. The collection method was also included as a covariate in the model to adjust for differences in species abundance across sites due to mosquito-biting behaviours. The number of person-times of capture was used as an offset to account for imbalanced sampling (because mosquitoes from 2 collection nights in one village were pooled prior to sorting, thereby impairing determination of specimen counts at the site level). Species with < 10 specimens were excluded from the analysis.

## Results

### Characteristics of the villages

The characteristics of the six villages are summarized in Additional file 1: Table S1. They were located on the forest hills of Karen state (mean elevation: 66 to 176 m, slope 3 to 13.4 m, proportion of forest 79 to 89%, cumulative length of rivers and streams: 0 to 183 km). The daily mean temperature collated by village was between 25.1 and 26 (range: 20.9–34.7) °C, and cumulative precipitation during the month before the survey was between 1357 and 1514 (range of daily values: 0 to 214) mm. The number of households was between 17 and 51 and the population size between 45 and 237.

### Epidemiological indicators of malaria and filariasis transmission

Overall annual falciparum and vivax malaria incidence rates were 0.40 (24/59488) and 0.96 (57/59488) cases per 1000 persons in 2019, and the range of monthly incidence rates was 0 to 10.70 and 0 to 16.04 cases per 1000 persons, respectively. The prevalence of lymphatic filariasis was 9.66% (66/683) and all detected cases were identified as *W. bancrofti* infections. The annual incidence of non-malaria fever was 7.50 cases per 1000 persons in 2019 (446/59488) and the range of monthly values was 0.76 to 26.74 cases per 1000 persons.

### Mosquito species diversity and biting behaviours

In total, 36,607 blood-seeking female mosquito specimens were collected and 96 species or species complexes in 16 genera were morphologically identified (Additional file 2: Table S2). Overall, *Anopheles* was the most abundant genus, followed by *Culex*, *Downsiomyia*, *Armigeres*, *Heizmannia*, *Aedes* and *Finlaya*. Other genera each accounted for < 1% of the collected specimens. Interestingly, the species count was less in indoor human landing catches and animal-baited traps than in outdoor human landing catches. The total number of captured mosquitoes varied between 1800 in village D and 11,271 in village A, the species count varied between 60 in village D and 76 in village B, and the resulting sample coverage estimate was > 0.98 in all villages (Table 1). The equal-coverage Hill richness (which provides higher leverage to rare taxa) varied between 31.7 (95% confidence interval [CI]: 29.7–33.7) in village A and 59.5 (95% CI: 42.8–76.2) in village D. The equal-coverage Hill-Shannon diversity (which emphasizes neither rare nor common taxa) and equal-coverage Hill-Simpson diversity (which provides higher leverage to common taxa) varied between 4.5 (95% CI: 4.3–4.6) and 21.8 (95% CI: 21–22.6) and between 2.9 (95% CI: 2.9–3) and 14.1 (95% CI: 13.4–14.7) in village F and village E, respectively. The biting behaviours of the mosquito species identified in this study are summarized in Additional file 3: Table S3.

**Table 1:** Summary of Hill diversity indices

Village	Sample size	Species count	Sample coverage	Equal-coverage Hill-Richness (95% CI)	Equal-coverage Hill-Shannon (95% CI)	Equal-coverage Hill-Simpson (95% CI)
A	11,271	64	0.9989	31.7 (29.7–33.7)	9.1 (8.9–9.3)	6.6 (6.4–6.7)
B	4649	76	0.9963	56.9 (51.5–62.4)	10.6 (10.1–11.1)	4.8 (4.6–5)
C	5367	64	0.9989	48.3 (45.7–50.9)	14.1 (13.6–14.5)	8.2 (7.9–8.5)
D	1800	60	0.9894	59.5 (43.5–75.5)	15.4 (14.4–16.4)	8.9 (8.3–9.5)
E	3433	71	0.9959	56.9 (53.1–60.7)	21.8 (20.7–22.9)	14.1 (13.3–14.8)
F	9756	72	0.9983	40.9 (37.6–44.2)	4.5 (4.3–4.6)	2.9 (2.8–3)

*CI* confidence interval

**Fig. 1:**
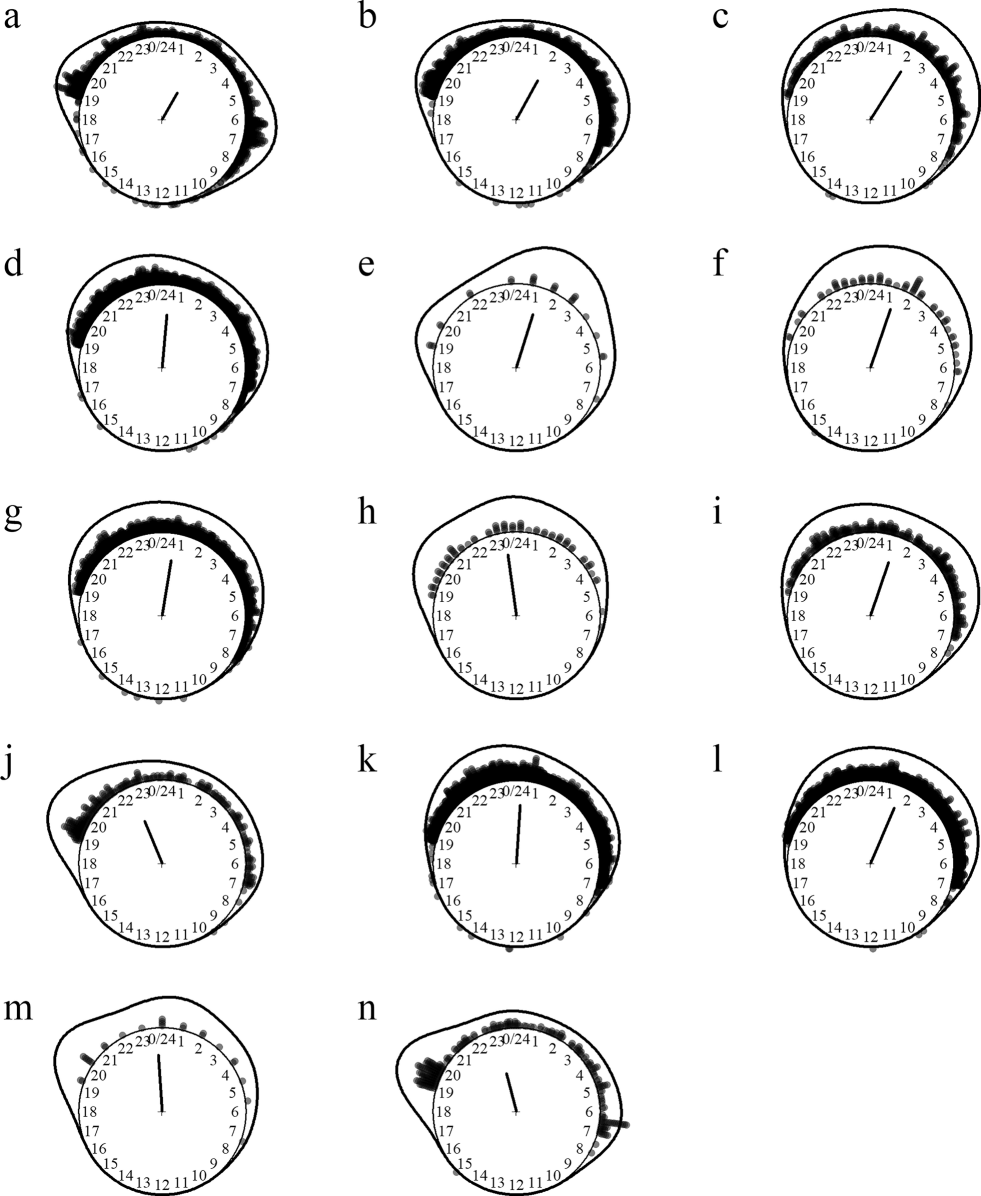
Biting time of *Anopheles* mosquito species. **a** *Anopheles (Ano.) barbirostris*, **b** *An. (Ano.) hyrcanus*, **c** *An. (Cel.) aconitus*, **d** *An. (Cel.) annularis*, **e** *An. (Cel.) culicifacies*, **f** *An. (Cel.) dirus*, **g** *An. (Cel.) jamesii*, **h** *An. (Cel.) jeyporiensis*, **i** *An. (Cel.) karwari*, **j** *An. (Cel.) kochi*, **k** *An. (Cel.) maculatus*, **l** *An. (Cel.) minimus*, **m** *An. (Cel.) tessellatus*, **n** *An. (Cel.) vagus*. The *dots* and *line* around the clock show the observed biting event times and kernel distribution under the circular probability model, respectively. The direction and length of the clock hand represent the von Mises distribution mean and concentration parameter estimates output by the model (see “Methods” section)

Fourteen *Anopheles* species of the subgenera *Anopheles* and *Cellia* were identified, including efficient malaria vectors (e.g. *An. dirus*, *An. maculatus* and *An. minimus*) and suspected vectors of lymphatic filariasis and Japanese encephalitis (e.g. *Anopheles annularis*). The species most aggressive to humans were *An. maculatus*, *An. minimus* and *An. annularis*. *Anopheles* mosquitoes preferentially fed on cattle over humans, as shown by the high cow- to outdoor human-biting rate ratios. *Anopheles culicifacies*, *An. dirus* and *An. vagus* were predominantly endophagous, whereas other species were predominantly exophagous. *Anopheles* mosquitoes were nighttime feeders starting to bite at dawn and resting after dusk (Fig. 1). Some species were also active in the morning and during late afternoon. Interestingly, *Anopheles barbirostris*, *An. hyrcanus*, *An. aconitus*, *An. minimus* and *An. maculatus* were active during daytime, albeit with low biting rates.

**Fig. 2:**
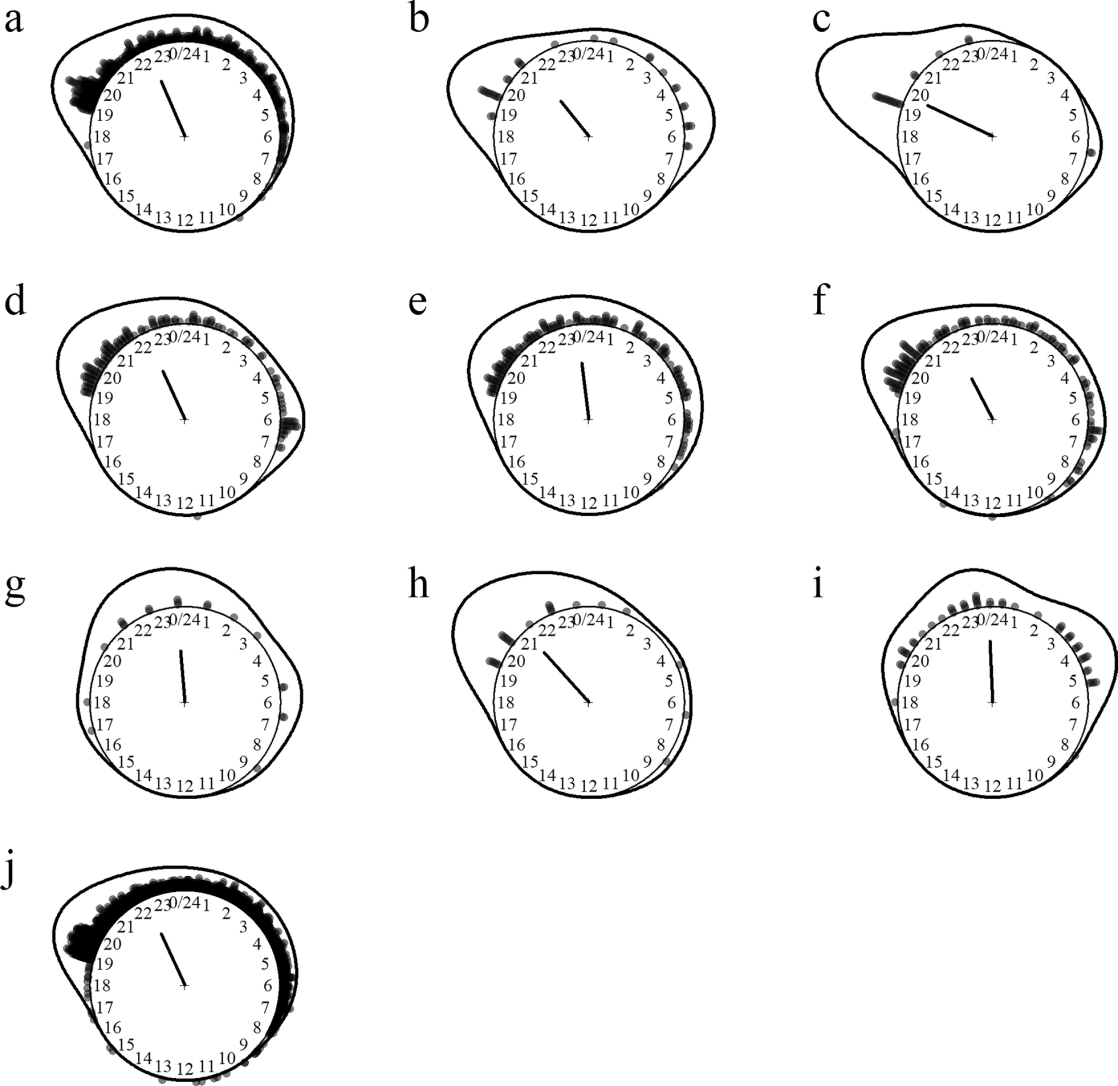
Biting time of *Culex* mosquito species. **a** *Culex (Cux.) alis*, **b** *Cx. (Cux.) fuscocephala*, **c** *Cx. (Cux.) sitiens*, **d** *Cx. (Cux.) tritaeniorhynchus*, **e** *Cx. (Cux.) vishnui*, **f** *Cx. (Cux.) whitmorei*, **g** *Cx. (Eum.) brevipalpis/phangngae*, **h** *Cx. (Lop.) mammilifer*, **i** *Cx. (Ocu.) bitaeniorhynchus*, **j** *Cx. (Ocu.) sinensis*. The *dots* and *line* around the clock show the observed biting event times and kernel distribution under the circular probability model, respectively. The direction and length of the clock hand represent the von Mises distribution mean and concentration parameter estimates output by the model (see “Methods” section)

Twenty-two species of five *Culex* subgenera were identified including the main vectors of Japanese encephalitis (*Culex tritaeniorhynchus*, *Cx. vishnui*, *Cx. gelidus* and *Cx. fuscocephala*) and suspected lymphatic filariasis vectors (*Culex bitaeniorhyncus* and *Cx. sitiens*). Noticeably, *Cx. quinquefasciatus* was not detected in these surveys. The species most aggressive to humans was by far *Culex sinensis*, followed by *Cx. alis* and *Cx. whitmorei*. *Culex* mosquitoes preferentially fed on cattle over humans and were variably exophagous. They were nighttime feeders collected almost exclusively between 7 p.m. and 9 a.m.; *Cx. whitmorei* and *Cx. sinensis* were seldom collected during daytime (Fig. 2).

**Fig. 3:**
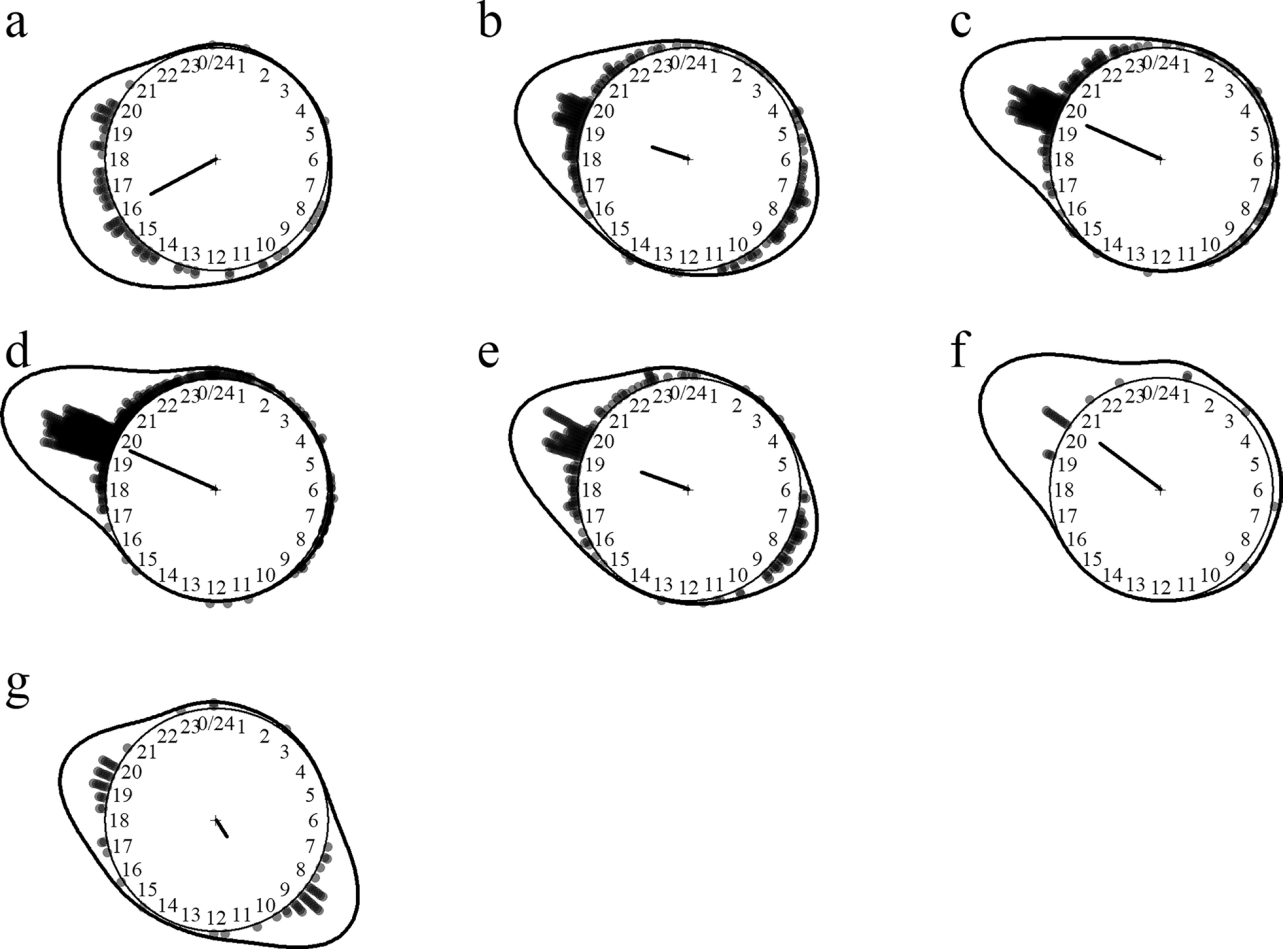
Biting time of *Downsiomyia* mosquito species. **a** *Downsiomyia ganapathi*, **b** *Do. harinasutai*, **c** *Do. mikrokopion*, **d** *Do. niveoides*, **e** *Do. novonivea*, **f** *Do. pexa/vana*, **g** *Do.* species 1. The *dots* and *line* around the clock show the observed biting event times and kernel distribution under the circular probability model, respectively. The direction and length of the clock hand represent the von Mises distribution mean and concentration parameter estimates outputted by the model (see “Methods” section)

Eight *Downsiomyia* species were identified including efficient lymphatic filariasis and dengue vectors (e.g. *Do. harinasutai* and *Do. niveoides*). The ones most aggressive to humans were *Downsiomyia niveoides*, *Do. mikrokopion* and *Do. harinasutai*. *Downsiomyia* mosquitoes preferred biting humans over cattle and were predominantly endophagous. These species had activity peaks at dusk and dawn with the notable exception of *Downsiomyia ganapathi*, which was a daytime feeder (Fig. 3).

**Fig. 4:**
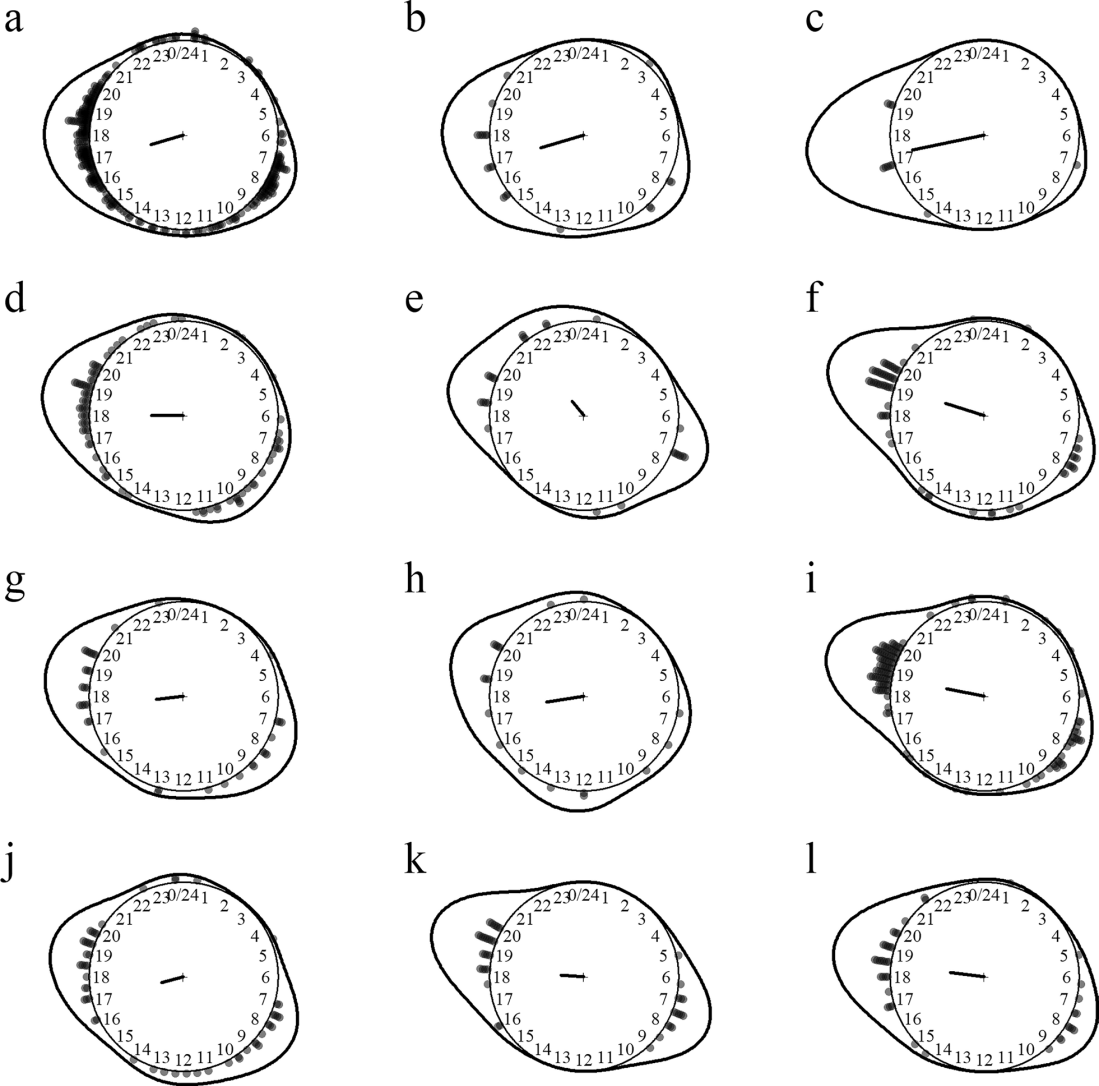
Biting time of *Armigeres* mosquito species. **a** *Armigeres (Arm.) kesseli*, **b** *Ar. (Arm.) kuchingensis*, **c** *Ar. (Arm.) moultoni*, **d** *Ar. (Arm.) subalbatus*, **e** *Ar. (Lei.) annulitarsis*, **f** *Ar. (Lei.) balteatus*, **g** *Ar. (Lei.) digitatus*, **h** *Ar. (Lei.) dolichocephalus*, **i** *Ar. (Lei.) flavus*, **j** *Ar. (Lei.) inchoatus*, **k** *Ar. (Lei.) magnus*, **l** *Ar. (Lei.) traubi*. The *dots* and *line* around the clock show the observed biting event times and kernel distribution under the circular probability model, respectively. The direction and length of the clock hand represent the von Mises distribution mean and concentration parameter estimates outputted by the model (see “Methods” section)

*Armigeres* was a diverse genus with nine species of the subgenus *Armigeres* and 11 species of the subgenus *Leicesteria*, including filariasis vectors and suspected vectors of Japanese encephalitis and Zika viruses (e.g. *Armigeres flavus* and *Ar. subalbatus*). The species most aggressive to humans were *Armigeres kesseli*, *Ar. flavus* and *Ar. subalbatus*. *Armigeres* mosquitoes preferentially fed on humans over cattle and were variably exophagous. These species were daytime feeders with activity peaks at dusk and dawn (Fig. 4).

**Fig. 5:**
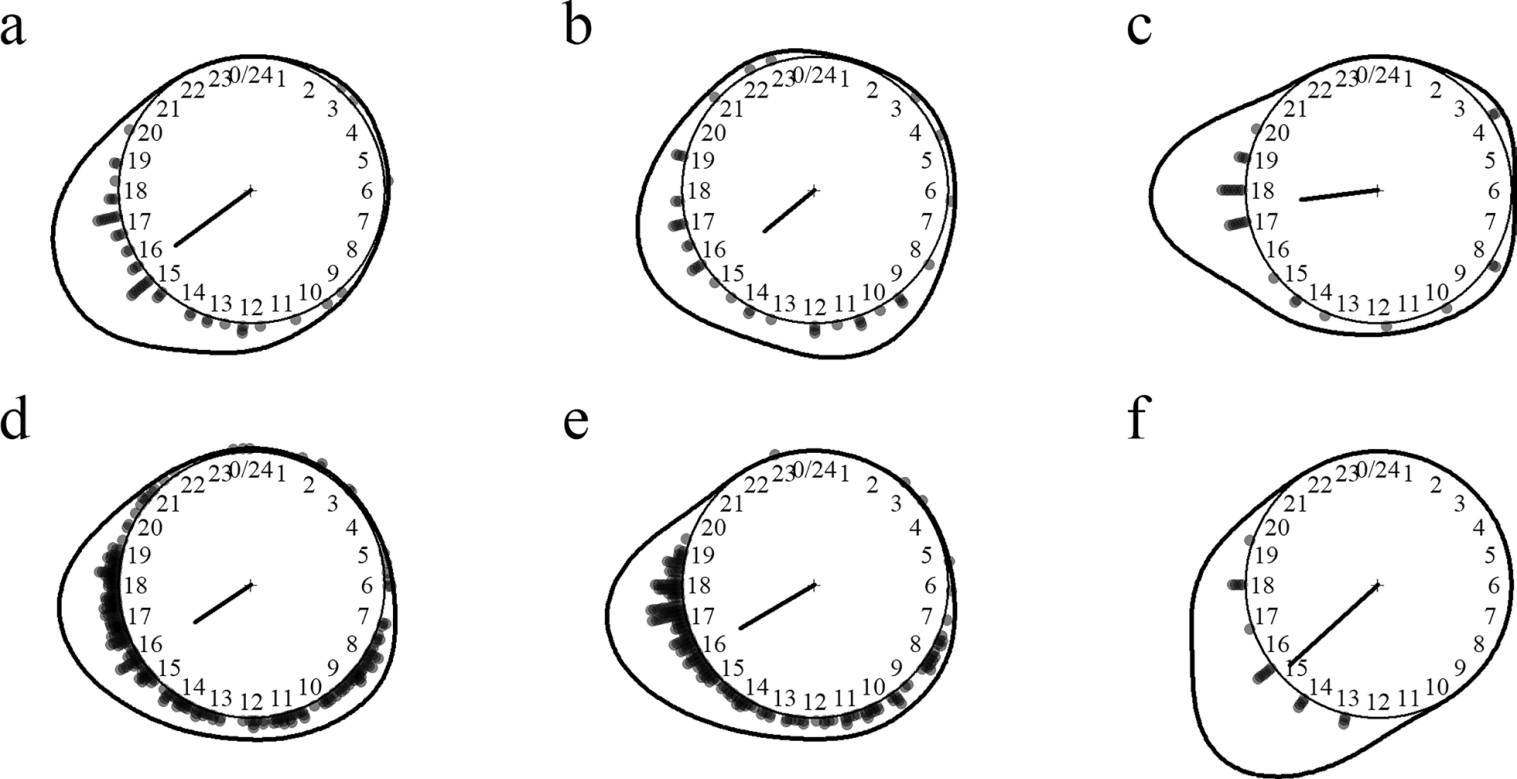
Biting time of *Heizmannia* mosquito species. **a** *Heizmannia (Hez.) aureochaeta*, **b** *Hz. (Hez.) chengi*, **c** *Hz. (Hez.) mattinglyi*, **d** *Hz. (Hez.) reidi*, **e** *Hz. (Hez.) scintillans*, **f** *Hz. (Mat.) catesi*. The *dots* and *line* around the clock show the observed biting event times and kernel distribution under the circular probability model, respectively. The direction and length of the clock hand represent the von Mises distribution mean and concentration parameter estimates outputted by the model (see “Methods” section)

*Heizmannia* represented only a small fraction of the overall number of collected specimens, but they had a strong preference for feeding on humans over cattle and relatively high human-biting rate maxima. Eight species of two subgenera were detected, but identification was particularly challenging in this genus. The species most aggressive to humans were *Heizmannia reidi*, *Hz. scintillans* and *Hz. aureochaeta*. These species were variably exophagous and active between 6 a.m. and 11 p.m. (Fig. 5).

**Fig. 6:**
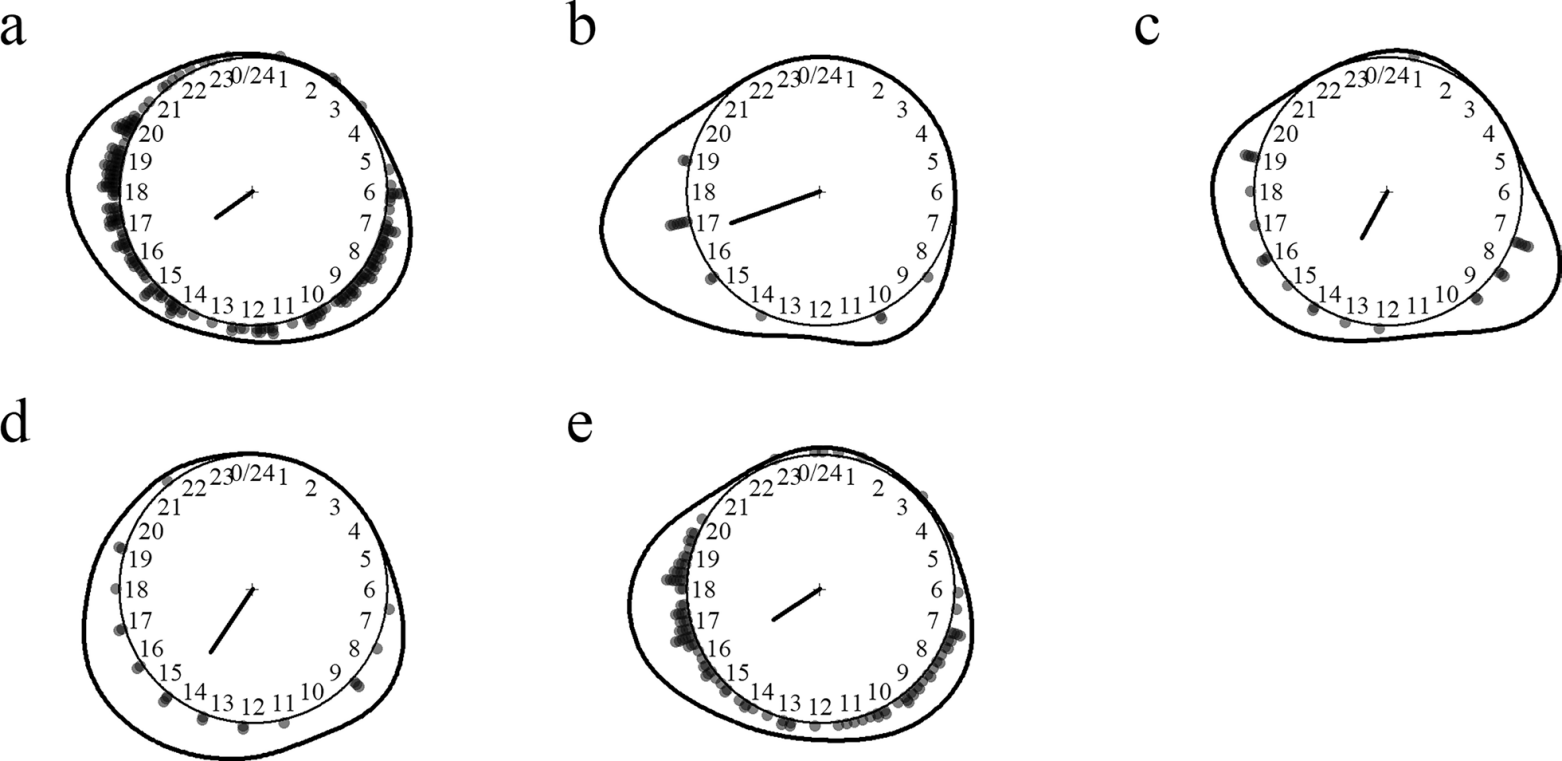
Biting time of *Aedes* mosquito species. **a** *Aedes* (*Stg*.) *albopictus*, **b** *Ae.* (*Stg*.) *annandalei*, **c** *Ae.* (*Stg*.) *desmotes*, **d** *Ae.* (*Stg*.) *malikuli*, **e** *Ae.* (*Stg*.) *pseudoalbopictus*. The *dots* and *line* around the clock show the observed biting event times and kernel distribution under the circular probability model, respectively. The direction and length of the clock hand represent the von Mises distribution mean and concentration parameter estimates outputted by the model (see “Methods” section)

Seven *Aedes* species were identified including important chikungunya, dengue and Zika viruses and lymphatic filariasis vectors (e.g. *Ae. albopictus* and *Ae. desmotes*). The species most aggressive to humans were *Aedes albopictus*, *Ae. pseudoalbopictus* and *Ae. desmotes*. Noticeably, *Ae. aegypti* was not detected in these surveys. *Aedes* mosquitoes preferred to feed on humans over cattle and were moderately exophagous. These species were predominantly daytime feeders active between 6 a.m. and 11 p.m. (Fig. 6).

**Fig. 7:**
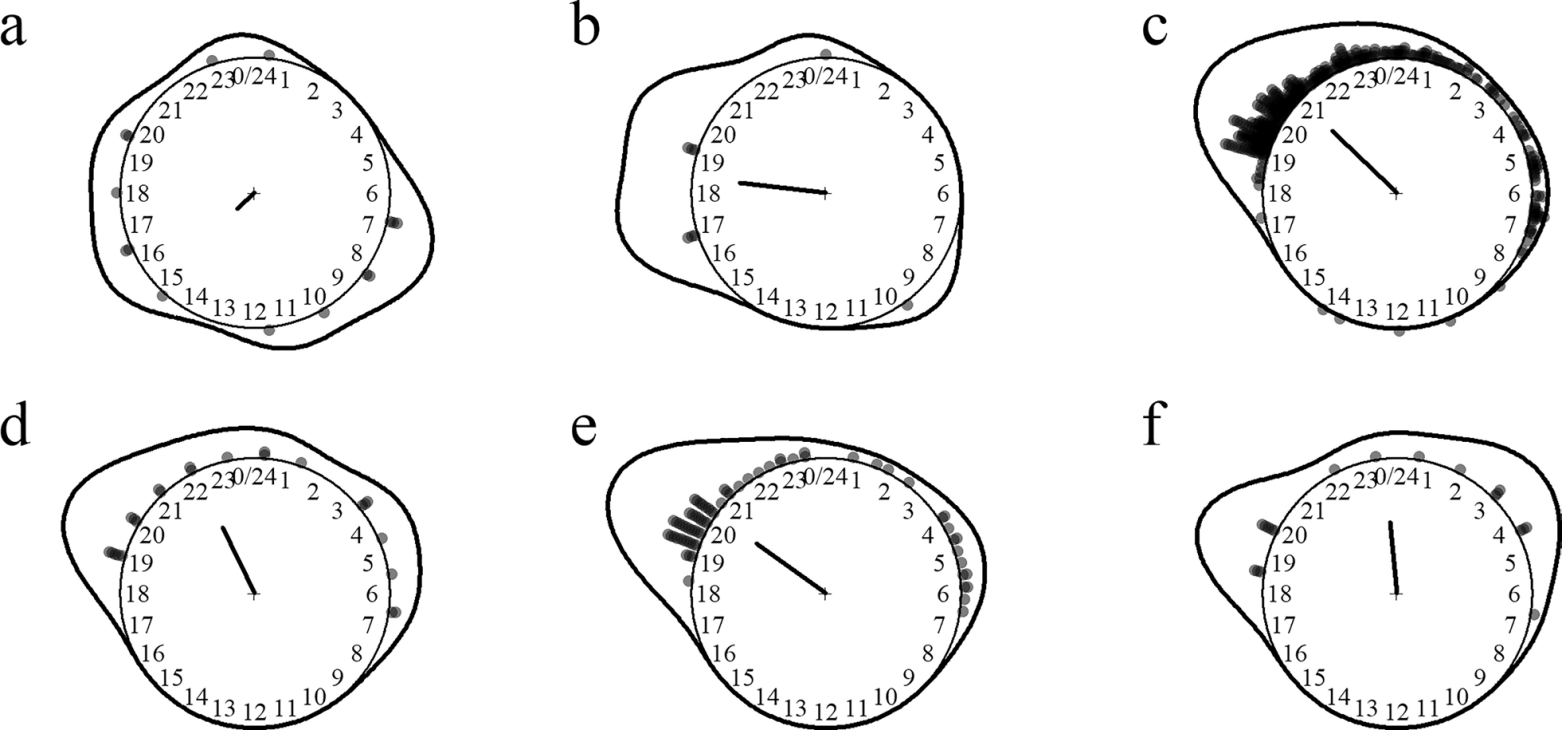
Biting time of other mosquito species. **a** *Bothaella eldrigei*, **b** *Danielsia albotaeniata*, **c** *Finlaya poicilia*, **d** *Mansionia* (*Mansonioides*) *uniformis*, **e** *Mansonia* (*Mansonioides*) *annulata*, **f** *Petermattinglyius* (*Petermattinglyius*) *iyengari*. The *dots* and *line* around the clock show the observed biting event times and kernel distribution under the circular probability model, respectively. The direction and length of the clock hand represent the von Mises distribution mean and concentration parameter estimates outputted by the model (see “Methods” section)

Other mosquito genera included 13 species of 8 other genera of the Aedinii tribe, 3 species of the genus *Mansonia* (Mansoinii tribe) and *Mimomyia fusca* (Facalbii tribe). The most abundant ones were *Finlaya poicila* and *Mansonia annulata*, two important lymphatic filariasis vectors. These species were nighttime feeders except for *Bothaella eldrigei* and *Danielsia albotaeniata*, which were active during daytime (Fig. 7). Interestingly, *Petermatinglyius* species were collected only with the human landing catch collection method.

### Mosquito species community assembly

Multivariate mosquito count data were modelled with a negative binomial generalized linear latent variable model accounting for overdispersion and residual between-species correlation (co-occurrence patterns, see Methods). The model fitted well the data as shown by the diagnostic plots (Additional file 4: Fig. S1 and Additional file 5: Fig. S2). Mosquito catching sites using the same collection method and of the same village clustered together in model-based ordination performed without including environmental variables (Additional file 6: Fig. S3). Examination of the trace of the residual covariance matrix before and after environmental variables were included in the model showed that environmental variables explained 85% of the variations in the data. The effects of environmental variables on species abundance are presented in Additional file 7: Fig. S4, and the residual covariance matrix of the model fitted with environmental variables, showing species co-occurrence patterns after controlling for covariation in species explained by the environmental terms, is presented in Additional file 8: Fig. S5.

The analysis revealed two clusters of species positively correlated with each other and negatively correlated with that of the other cluster. The first cluster included *Culex* (*Cx. alis*, *Cx. fuscocephala*, *Cx. sinensis*, *Cx. tritaeniorhynchus*, *Cx. vishnui* and *Cx. whitmorei*) and *Anopheles* (*An. aconitus*, *An. annularis*, *An. hyrcanus*, *An. jamesi*, *An. karwari*, *An. kochi*, *An. maculatus*, *An. minimus* and *An. vagus*) species, and *Ma. annulata*, which typically breed in shallow, stagnant or slow-moving water, such as marshes, swamps, rice fields and the margins of streams and puddles. Overall, these species were negatively associated with elevation, slope, forest (except *Anopheles karwari*, which was strongly associated with sparse forest) and positively associated with grasslands, shrubs and crop fields.

The second cluster included many rainforest mosquitoes of the genera *Heizmannia* (*Hz. aureochaeta*, *Hz. chengi*, *Hz. scintillans* and *Hz. reidi*), *Downsiomyia* (*Do. ganapathi*, *Do. harinasutai*, *Do. niveoides*, *Do. novonivea*, *Do. mikrokopion* and *Do*. species 1) and *Armigeres* (*Ar. balteatus*, *Ar. digitatus*, *Ar. flavus*, *Ar. inchoatus*, *Ar. kesselli*, *Ar. magnus*, *Ar. subalbatus* and *Ar. traubi*), *Anopheles barbirostris*, *An. dirus*, *An. jeyporiensis*, *Cx. bitaeniorhyncus*, *Fl. poicilia* and *Ae. pseudoalbopictus*, which typically breed in tree canopy and natural surface water and containers such as bamboo stumps, tree holes and rainwater pools. Overall, these species were positively associated with elevation, slope, dense forest, surface water and wetlands and negatively associated with crop fields, grasslands and shrubs.

Deforestation had mixed effects on vector abundance. It was positively associated with species striving in sparse forest (e.g. *Anopheles dirus*, *An. karwari* and *Ar. inchoatus*) and negatively associated with species striving in dense forest (*An. jeyporiensis*, *Ar. balteatus* and *Ar. traubi*). *Anopheles aconitus*, *Cx. fuscocephala*, *Cx. vishnui*, *Cx. whitmorei* and *Ma. annulata* were negatively associated with both forest and deforestation. Dense plantation was positively associated with *An. jeyporiensis*, *An. karwari* and *Ar. balteatus* but negatively associated with *Ar. traubi*. Interestingly, *Ae. albopictus* was not correlated with other species and only weakly or not associated with the environmental variables included in this analysis.

## Discussion

We report the results of the first systematic study to our knowledge of *Culicidae* species communities biting humans and their livestock in the forest hills of Karen state, and one of the very few such studies conducted in Southeast Asia. The diversity and biting behaviours of medically important malaria, arboviruses and lymphatic filariasis vectors were described together with the patterns of species co-occurrence and effects of environmental variables on vector abundance. The results are consistent with existing knowledge on the ecology and biology of species endemic to this area [34–39] and provide new information on their biting behaviours and species community assembly.

Two different species community profiles corresponding to rice-breeding and rainforest mosquitoes were identified. Rice field ecotype was associated with culicine vectors of Japanese encephalitis, whereas rainforest was positively associated with *Armigeres*, *Downsiomyia* and *Aedes* species transmitting dengue, chikungunya, Zika and lymphatic filariasis. As expected, malaria vectors were found in both assembly profiles (the villages were surveyed because they had high burdens of malaria) but the species were different. Deforestation had a mixed effect on vector abundance and was positively associated with the main local malaria vectors. This could be explained by the effects of increased availability of breeding sites and blood resources resulting from anthropogenic activities on some relatively well domesticated species [49]. Dense plantation appeared to be a suitable habitat for some forest mosquito species, whereas crop fields, grasslands and shrubs were positively associated with rice-breeding species. Therefore, deforestation and intensification of agricultural practice may be of relevance to the ecology and evolution of mosquito-borne diseases in this region. *Aedes albopictus* did not display a specific co-occurrence pattern or strong association with environmental variables, suggesting the role of ecological plasticity or cryptic diversity in driving the widespread distribution of this important vector species [50, 51].

The implications of vector bionomics for the efficacy of vector control is another key finding of this study. In this area, mosquito bed nets only have a marginal impact on disease transmission [52] because relevant vector species bite outdoors with activity peaks at dusk and dawn or during the daytime [5, 53, 54]. Similarly, outdoor resting challenges the efficacy of indoor residual spraying [55, 56]. Personal protection with long-sleeved clothes and skin repellents is often the only option available, but the efficacy is limited and it is difficult to implement programmatically [57, 58]. Outdoor residual spraying of the peridomestic vegetation and forest fringe can quickly decrease vector densities, biting rates and longevity, but it is difficult to deploy, toxic to the environment and increases vector resistance [59]. Therefore, it can probably be used for outbreak response to quickly interrupt transmission in a few places but not for routine vector control over large spatial scales. Zoophagous and opportunistic blood type selection contribute to the limited effects of mosquito bed nets on vector populations [5, 54] but open avenues for veterinary-based interventions such as treatment of livestock with long-lasting formulations of endectocides or topical application of residual insecticides [60]. The feasibility of larval source management depends on larval habitats. Unlike in urban or peri-urban settings, natural breeding sites in these forested areas are too diverse, fragmented or difficult to access for removal or treatment with insecticides [61]. One exception is rice breeding species (including many culicine and some anopheline vectors), which can be targeted with larvicides or biological control agents such as larvivorous fishes [62]. However, these species are often exposed to agrochemicals and develop high levels of resistance to multiple insecticides, warranting further investigation [63].

This study has several limitations. The number of villages was small, villages were in a relatively small geographic area and collection sites were only set up within or near the village. Therefore, this study may not be representative of the variety of habitats found in Karen state. Furthermore, the single cross-sectional design could not capture seasonality. No molecular identification was performed, and sibling species were not assessed; hence, cryptic diversity and misidentification are potential confounders in this study. Blood-type preferences were determined by comparing human- and cow-biting rates. Other relevant animal species (e.g. birds and pigs) were not assessed, thereby preventing comprehensive characterization of zoophagous biting behaviours. Land use-land cover maps had relatively inaccurate categories and low resolution regardin the understanding of mosquito ecology. For example, although sparse and dense forests could be distinguished, current classification was not in line with the variety of forest types found in this area [64]. Similarly, crop field and plantation types were not determined, and there was no specific category for rice fields. Surface water habitats under the forest canopy could not be characterized. Only blood-seeking females were collected, and important knowledge gaps remain in vector bionomics. Studies of other life history traits (e.g. resting, mating and plant feeding), development stages (e.g. larvae and males) and insecticide resistance patterns should be performed to guide the development of more effective vector control interventions.

Future research is warranted to scale up entomological surveillance and better understand the relationship between mosquitoes and the environment. This would require developing efficient trapping methods and high-throughput molecular assays for species and blood meal identification, pathogen detection and age grading. Studies aiming to improve environmental characterization and develop environment-based prediction models of vector abundance to identify high-risk areas and target interventions can also be undertaken. Evaluation of vector control interventions addressing outdoor and early mosquito biting exposures would be particularly valuable to disease control and elimination programmes [65]. This would require detailed knowledge not only of vector bionomics and resistance patterns but also of the complex behavioural interactions among humans, animals and mosquitoes that determine actual vector biting exposures and intervention effects on vector populations [66]. Assessment of antibody responses directed against mosquito salivary components has been proposed as an outcome measure of vector biting exposures, but this approach needs further validation before it can be deployed in the field [67].

## Conclusions

The transmission dynamics of mosquito-borne diseases are particularly complex in forested areas of the Thailand-Myanmar border. Environmental factors shape the assembly of mosquito species communities and largely determine the risk of exposure to vector bites. Future research is needed to better characterize vector bionomics across rural Southeast Asia and develop vector control interventions effective against outdoor and early biters.

## Author contributions

TK, NG and NJ processed the mosquito samples. SS supervised the laboratory teams and cured the data. CP wrote analysis code and provided data. FG and VH developed the methodology, contributed to funding acquisition, wrote analysis code and provided data. VC designed the study, contributed to funding acquisition, developed the methodology and wrote analysis code, processed the mosquito samples, performed the analysis and data visualization, cured the data, supervised the laboratory teams, administered the project and wrote the original draft of the manuscript. FN designed the study, led funding acquisition, administered the project, and reviewed and edited the manuscript. All authors read and approved the final manuscript.

## Supplementary Information

Additional file 1: Table S1. Demographic, epidemiological and environmental characteristics of the villages.

Additional file 2: Table S2. Summary of Culicidae diversity.

Additional file 3: Table S3. Biting behaviours of the mosquito species identified in this study.

Additional file 4: Figure S1. Diagnostic plots for the negative binomial generalized linear latent variable model without environmental variables.

Additional file 5: Figure S2. Diagnostic plots for the negative binomial generalized linear latent variable model with environmental variables.

Additional file 6: Figure S3. Ordination plot with 30 indicator species based on the negative binomial generalized linear latent variable model fitted to the mosquito data without environmental variables.

Additional file 7: Figure S4. Plots of the point estimates (ticks) for coefficients of the environmental variables and their 95% confidence intervals (lines) for the negative binomial generalized linear latent variable model, with those coloured in grey (black) denoting intervals (not) containing zero.

Additional file 8: Figure S5. Residual correlation matrix based on latent factor loadings for the negative binomial generalized linear latent variable model with environmental covariates.

## Data Availability

All data generated or analysed during this study are included in this published article and its supplementary information files.

## Abbreviations

CI: Confidence interval
RT-qPCR: Quantitative reverse transcription polymerase chain reaction
RNA: Ribonucleic acid
NDVI: Normalized Difference Vegetation Index
MNDWI: Modified Normalized Difference Water Index
NDWIGAO: Gao’s Normalized Difference Water Index
