# Supplementary material for: Assessment of mosquito species communities biting humans and their livestock in the forest hills of Karen state, Myanmar: a cross-sectional survey in six villages
Source: Parasit Vectors. 2025 Dec 29;19:58. doi: 10.1186/s13071-025-07217-9 (PMC12860035; doi:10.1186/s13071-025-07217-9)
Supplement: Supplementary file 1 — Additional file 1: Table S1. Demographic, epidemiological and environmental characteristics of the villages. [file 13071_2025_7217_MOESM1_ESM.docx]

Table S1 Demographic, epidemiological and environmental characteristics of the villages.

| Characteristic | Village | | | | | |
| --- | --- | --- | --- | --- | --- | --- |
|  | A | B | C | D | E | F |
| Demographics |  |  |  |  |  |  |
| Number of households | 17 | 51 | 35 | 30 | 27 | 48 |
| Population Size | 106 | 142 | 45 | 113 | 135 | 237 |
| Sex |  |  |  |  |  |  |
| Female | 46 (43%) | 77 (54%) | 18 (40%) | 57 (50%) | 65 (48%) | 111 (47%) |
| Male | 60 (57%) | 65 (46%) | 27 (60%) | 56 (50%) | 70 (52%) | 119 (50%) |
| Undetermined | 0 (0%) | 0 (0%) | 0 (0%) | 0 (0%) | 0 (0%) | 7 (3%) |
| Mean age (years) | 27.9 (19.1) | 23.1 (19.7) | 37.9 (13.5) | 19.2 (16.7) | 21.9 (18.3) | 22.4 (19.3) |
| Epidemiological indicators |  |  |  |  |  |  |
| Annual falciparum malaria incidence (per 1000 persons) [range of monthly rates] | 0.103 (4/38.69) [0 to 1.258] | 0.27 (14/51.83) [0 to 2.113] | 0 (0/16.425) | 0.048 (2/41.245) [0 to 0.295] | 0 (0/49.275) | 0.046 (4/86.505) [0 to 0.408] |
| Annual vivax malaria incidence (per 1000 persons) [range of monthly rates] | 0.258 (10/38.69) [0 to 1.887] | 0.232 (12/51.83) [0 to 0.909] | 0.365 (6/16.425) [0 to 2.151] | 0.17 (7/41.245) [0 to 0.632] | 0 (0/49.275) | 0.277 (24/86.505) [0 to 0.681] |
| Prevalence of submicroscopic *P. falciparum* infections | 12/82 (14.6%) | 20/66 (30.3%) | 4/46 (8.7%) | 1/44 (2.3%) | 14/72 (19.4%) | 30/116 (25.9%) |
| Prevalence of submicroscopic *P. vivax* infections | 20/82 (24.4%) | 25/66 (37.9%) | 19/46 (41.3%) | 20/44 (45.5%) | 25/72 (34.7%) | 35/116 (30.2%) |
| Prevalence of *W. bancrofti* microfilaremia | 1/82 (1.2%) | 5/59 (8.5%) | 1/45 (2.2%) | 2/44 (4.5%) | 5/66 (7.6%) | 30/109 (27.5%) |
| Annual non-malaria fever incidence (per 1000 persons) [range of monthly rates] | 1.447 (56/38.69) [0.609 to 3.348] | 1.929 (100/51.83) [0.454 to 3.286] | 3.288 (54/16.425) [0.717 to 14.337] | 1.503 (62/41.245) [0.571 to 3.14] | 0.791 (39/49.275) [0.239 to 1.728] | 1.526 (132/86.505) [0.136 to 2.813] |
| Environmental variables |  |  |  |  |  |  |
| Mean elevation (m) | 113 | 126 | 66 | 91 | 66 | 176 |
| Mean slope (m) | 13.41 | 2.76 | 11.05 | 2.11 | 3.03 | 5.06 |
| Cumulative length of river and streams (km) | 3.22 | 17.34 | 0 | 5.88 | 183.47 | 16.70 |
| Hansen deforestation index | 0.69 | 1.05 | 2.41 | 3.48 | 2.76 | 1.37 |
| LULC class (%) |  |  |  |  |  |  |
| Bare areas | 0 | 0.4 | 0 | 0 | 1 | 0 |
| Built-up areas | 0 | 0 | 0 | 0 | 0.6 | 0 |
| Crop fields | 14.6 | 8.5 | 14.2 | 9.7 | 9.1 | 2 |
| Dense forests | 60 | 51.8 | 34.3 | 26 | 34.1 | 62.3 |
| Dense plantations | 0.6 | 2.9 | 2.1 | 1.7 | 1.6 | 2.3 |
| Grassland and shrubs | 3 | 6.4 | 4.1 | 6 | 4.4 | 6.1 |
| Roads | 1.9 | 0.9 | 0 | 1.5 | 0.6 | 0.6 |
| Sparse forests | 19.8 | 29.1 | 45.3 | 55.1 | 45 | 26.7 |
| Water areas | 0 | 0 | 0 | 0 | 3.4 | 0 |
| Wetlands | 0 | 0 | 0 | 0 | 0.2 | 0 |
| Monthly cumulative precipitation (mm) [range] | 1514 [0 to 214] | 1357 [0 to 152] | 1463 [0 to 173] | 1448 [0 to 170] | 1416 [0 to 211] | 1383 [0 to 166] |
| Daily mean temperature (°C) [range] | 25.1 [21.1 to 33.3] | 25.2 [20.9 to 33.5] | 26 [22 to 34.7] | 26 [22 to 34.7] | 25.1 [21.1 to 33.3] | 25.2 [20.9 to 33.5] |
| Daily mean dewpoint (°C) [range] | 23.3 [18.2 to 24.8] | 23.3 [18.2 to 24.6] | 24.1 [18.7 to 25.3] | 24.1 [18.7 to 25.3] | 23.3 [18.2 to 24.8] | 23.3 [18.2 to 24.6] |
| Mean NDVI | 0.638 (0.064) | 0.7 (0.017) | 0.705 (0.012) | 0.681 (0.01) | 0.718 (0.012) | 0.727 (0.027) |
| Mean MNDWI | -0.311 (0.09) | -0.4 (0.014) | -0.404 (0.012) | -0.378 (0.017) | -0.439 (0.021) | -0.434 (0.018) |
| Mean NDWIGAO | 0.259 (0.016) | 0.264 (0.021) | 0.26 (0.018) | 0.242 (0.026) | 0.248 (0.009) | 0.275 (0.019) |

*Abbreviations*: LULC, land use land cover; MNDWI, Modified Normalized Difference Water Index; NDVI, Normalized Difference Vegetation; NDWIGAO, Gao’s Normalized Difference Water Index; SD, standard deviation. Data are n (%), mean (SD), or n/N (%).
