# Supplementary material for: Assessment of mosquito species communities biting humans and their livestock in the forest hills of Karen state, Myanmar: a cross-sectional survey in six villages
Source: Parasit Vectors. 2025 Dec 29;19:58. doi: 10.1186/s13071-025-07217-9 (PMC12860035; doi:10.1186/s13071-025-07217-9)
Supplement: Supplementary file 2 — Additional file 2: Table S2. Summary of Culicidae diversity. [file 13071_2025_7217_MOESM2_ESM.docx]

Table S2 Overview of *Culicidae* diversity.

| Genus | Indoor human-landing catch | | | | Outdoor human-landing catch | | | | Animal-baited trap | | | | Overall | | | |
| --- | --- | --- | --- | --- | --- | --- | --- | --- | --- | --- | --- | --- | --- | --- | --- | --- |
|  | Species count | Specimen count | Relative abundance (%) | Rank | Species count | Specimen count | Relative abundance (%) | Rank | Species count | Specimen count | Relative abundance (%) | Rank | Species count | Specimen count | Relative abundance (%) | Rank |
| *Anopheles* | 14 | 2197 | 41.52 | 1 | 14 | 6529 | 61.27 | 1 | 14 | 17097 | 85.04 | 1 | 14 | 25823 | 70.54 | 1 |
| *Culex* | 14 | 1278 | 24.15 | 2 | 13 | 2028 | 19.03 | 2 | 15 | 2463 | 12.25 | 2 | 22 | 6263 | 17.11 | 2 |
| *Downsiomyia* | 7 | 968 | 18.3 | 3 | 8 | 840 | 7.88 | 3 | 8 | 177 | 0.88 | 3 | 8 | 2009 | 5.49 | 3 |
| *Armigeres* | 17 | 254 | 4.8 | 4 | 19 | 463 | 4.34 | 4 | 16 | 127 | 0.63 | 4 | 20 | 857 | 2.34 | 4 |
| *Heizmannia* | 7 | 240 | 4.54 | 5 | 8 | 361 | 3.39 | 5 | 5 | 25 | 0.12 | 7 | 8 | 626 | 1.71 | 5 |
| *Finlaya* | 1 | 151 | 2.85 | 6 | 1 | 116 | 1.09 | 7 | 2 | 120 | 0.6 | 5 | 2 | 399 | 1.09 | 6 |
| *Aedes* | 7 | 137 | 2.59 | 7 | 7 | 236 | 2.21 | 6 | 5 | 20 | 0.1 | 8 | 7 | 395 | 1.08 | 7 |
| *Mansonia* | 3 | 28 | 0.53 | 8 | 2 | 33 | 0.31 | 8 | 3 | 59 | 0.29 | 6 | 3 | 125 | 0.34 | 8 |
| *Petermattinglyius* | 2 | 9 | 0.17 | 9 | 3 | 17 | 0.16 | 9 | 0 | 0 | 0 | 15 | 3 | 28 | 0.08 | 9 |
| *Bothaella* | 1 | 3 | 0.06 | 12 | 1 | 11 | 0.1 | 10 | 1 | 1 | 0 | 11 | 1 | 15 | 0.04 | 10 |
| *Hulecoeteomyia* | 2 | 5 | 0.09 | 10 | 3 | 3 | 0.03 | 11 | 1 | 3 | 0.01 | 9 | 3 | 11 | 0.03 | 11 |
| *Danielsia* | 1 | 4 | 0.08 | 11 | 1 | 3 | 0.03 | 12 | 1 | 1 | 0 | 12 | 1 | 10 | 0.03 | 12 |
| *Ayurakitia* | 0 | 0 | 0 | 15 | 1 | 3 | 0.03 | 13 | 0 | 0 | 0 | 14 | 1 | 4 | 0.01 | 13 |
| *Mimomyia* | 0 | 0 | 0 | 13 | 1 | 2 | 0.02 | 14 | 1 | 2 | 0.01 | 10 | 1 | 4 | 0.01 | 14 |
| *Frewardsius* | 0 | 0 | 0 | 14 | 0 | 0 | 0 | 16 | 1 | 1 | 0 | 13 | 1 | 2 | 0.01 | 15 |
| *Phagomyia* | 0 | 0 | 0 | 16 | 1 | 1 | 0.01 | 15 | 0 | 0 | 0 | 16 | 1 | 1 | 0 | 16 |
| Undetermined | 0 | 17 | 0.32 | NA | 0 | 10 | 0.09 | NA | 0 | 8 | 0.04 | NA | 0 | 35 | 0.1 | NA |
| Total | 76 | 5291 | NA | NA | 83 | 10656 | NA | NA | 73 | 20104 | NA | NA | 96 | 36607 | NA | NA |

*Abbreviations*: ABT, animal-baited trap; HLC, human-landing catch; NA, not applicable.
