# Supplementary material for: Assessment of mosquito species communities biting humans and their livestock in the forest hills of Karen state, Myanmar: a cross-sectional survey in six villages
Source: Parasit Vectors. 2025 Dec 29;19:58. doi: 10.1186/s13071-025-07217-9 (PMC12860035; doi:10.1186/s13071-025-07217-9)
Supplement: Supplementary file 3 — Additional file 3: Table S3. Biting behaviours of the mosquito species identified in this study. [file 13071_2025_7217_MOESM3_ESM.docx]

**Table S3** Biting behaviours of mosquito species identified in this study.

| Species | No. specimens | Mean indoor human-biting rate (no. bites/person/night) [range] | Mean outdoor human-biting rate (no. bites/person/night) [range] | Mean cow-biting rate (no. bites/person/night) [range] | Mean outdoor / indoor human-biting rate ratio | Mean cow / outdoor human-biting rate ratio |
| --- | --- | --- | --- | --- | --- | --- |
| *An.* (*Ano.*) *barbirostris* | 1090 | 0.36 [0 to 8] | 0.84 [0 to 12] | 32.75 [0 to 239] | 2.3 | 39.2 |
| *An.* (*Ano.*) *hyrcanus* | 3353 | 0.27 [0 to 8] | 0.4 [0 to 10] | 116.36 [4 to 813] | 1.5 | 290.9 |
| *An.* (*Cel.*) *aconitus* | 519 | 0.16 [0 to 3] | 0.51 [0 to 11] | 15.18 [0 to 127] | 3.3 | 29.5 |
| *An.* (*Cel.*) *annularis* | 3632 | 0.46 [0 to 7] | 2.36 [0 to 24] | 114.25 [11 to 477] | 5.1 | 48.5 |
| *An.* (*Cel.*) *culicifacies* | 25 | 0.03 [0 to 1] | 0.01 [0 to 1] | 0.68 [0 to 2] | 0.2 | 95 |
| *An.* (*Cel.*) *dirus* | 62 | 0.21 [0 to 4] | 0.09 [0 to 2] | 0.57 [0 to 3] | 0.4 | 6.2 |
| *An.* (*Cel.*) *jamesii* | 2028 | 0.15 [0 to 4] | 0.82 [0 to 25] | 65.93 [3 to 328] | 5.5 | 80.3 |
| *An.* (*Cel.*) *jeyporiensis* | 68 | 0.1 [0 to 2] | 0.14 [0 to 3] | 1.07 [0 to 10] | 1.4 | 7.9 |
| *An.* (*Cel.*) *karwari* | 349 | 0.09 [0 to 2] | 0.19 [0 to 4] | 10.96 [0 to 68] | 2.2 | 56.9 |
| *An.* (*Cel.*) *kochi* | 261 | 0.09 [0 to 3] | 0.14 [0 to 4] | 7.75 [0 to 30] | 1.7 | 54.2 |
| *An.* (*Cel.*) *maculatus* | 9021 | 3.71 [0 to 33] | 16.8 [0 to 125] | 135.54 [15 to 572] | 4.5 | 8.1 |
| *An.* (*Cel.*) *minimus* | 5086 | 6.14 [0 to 63] | 10.68 [0 to 90] | 57.93 [5 to 245] | 1.7 | 5.4 |
| *An.* (*Cel.*) *tessellatus* | 26 | 0.01 [0 to 1] | 0.02 [0 to 1] | 0.79 [0 to 4] | 3 | 36.7 |
| *An.* (*Cel.*) *vagus* | 231 | 0.03 [0 to 2] | 0.01 [0 to 1] | 8 [0 to 26] | 0.5 | 560 |
| *Ar.* (*Arm.*) *bhayungi* | 1 | 0 | 0 | 0.04 [0 to 1] | NA | Inf |
| *Ar.* (*Arm.*) *confusus* | 1 | 0 | 0.01 [0 to 1] | 0 | NA | NA |
| *Ar.* (*Arm.*) *jugraensis* | 6 | 0.02 [0 to 1] | 0.01 [0 to 1] | 0 | 0.7 | 0 |
| *Ar.* (*Arm.*) *kesseli* | 299 | 0.61 [0 to 6] | 1 [0 to 16] | 1.82 [0 to 11] | 1.6 | 1.8 |
| *Ar.* (*Arm.*) *kuchingensis* | 19 | 0.05 [0 to 2] | 0.06 [0 to 3] | 0.04 [0 to 1] | 1.3 | 0.6 |
| *Ar.* (*Arm.*) *malayi* | 2 | 0.01 [0 to 1] | 0.01 [0 to 1] | 0 | 1 | 0 |
| *Ar.* (*Arm.*) *moultoni* | 10 | 0.04 [0 to 3] | 0.02 [0 to 1] | 0 | 0.6 | 0 |
| *Ar.* (*Arm.*) *subalbatus* | 85 | 0.1 [0 to 3] | 0.32 [0 to 3] | 0.61 [0 to 5] | 3.2 | 1.9 |
| *Ar.* (*Arm.*) *theobaldi* | 7 | 0.01 [0 to 1] | 0.01 [0 to 1] | 0.07 [0 to 1] | 1 | 5 |
| *Ar.* (*Lei.*) *annulipalpis* | 7 | 0.03 [0 to 1] | 0.01 [0 to 1] | 0.04 [0 to 1] | 0.2 | 5 |
| *Ar.* (*Lei.*) *annulitarsis* | 24 | 0.04 [0 to 3] | 0.1 [0 to 2] | 0.14 [0 to 2] | 2.3 | 1.4 |
| *Ar.* (*Lei.*) *balteatus* | 65 | 0.09 [0 to 2] | 0.3 [0 to 6] | 0.11 [0 to 2] | 3.2 | 0.4 |
| *Ar.* (*Lei.*) *dentatus* | 2 | 0 | 0.01 [0 to 1] | 0.04 [0 to 1] | Inf | 5 |
| *Ar.* (*Lei.*) *digitatus* | 36 | 0.06 [0 to 2] | 0.14 [0 to 2] | 0.21 [0 to 3] | 2.1 | 1.6 |
| *Ar.* (*Lei.*) *dolichocephalus* | 18 | 0.06 [0 to 4] | 0.05 [0 to 2] | 0.07 [0 to 1] | 0.8 | 1.4 |
| *Ar.* (*Lei.*) *flavus* | 109 | 0.17 [0 to 4] | 0.47 [0 to 4] | 0.43 [0 to 3] | 2.8 | 0.9 |
| *Ar.* (*Lei.*) *inchoatus* | 55 | 0.13 [0 to 3] | 0.16 [0 to 3] | 0.11 [0 to 1] | 1.3 | 0.7 |
| *Ar.* (*Lei.*) *longipalpis* | 2 | 0.01 [0 to 1] | 0 | 0 | 0 | NA |
| *Ar.* (*Lei.*) *magnus* | 42 | 0.08 [0 to 2] | 0.15 [0 to 3] | 0.18 [0 to 1] | 1.9 | 1.2 |
| *Ar.* (*Lei.*) *traubi* | 41 | 0.13 [0 to 2] | 0.09 [0 to 2] | 0.14 [0 to 2] | 0.7 | 1.5 |
| *Ay. griffithi*/*peytoni* | 4 | 0 | 0.02 [0 to 1] | 0 | Inf | 0 |
| *Bo. eldrigei* | 15 | 0.02 [0 to 1] | 0.07 [0 to 2] | 0.04 [0 to 1] | 3.3 | 0.5 |
| *Cx.* (*Cux.*) *alis* | 564 | 0.42 [0 to 6] | 0.86 [0 to 13] | 12.93 [0 to 38] | 2 | 15.1 |
| *Cx.* (*Cux.*) *fuscocephala* | 31 | 0 | 0.03 [0 to 1] | 0.96 [0 to 3] | Inf | 33.8 |
| *Cx.* (*Cux.*) *gelidus* | 5 | 0.01 [0 to 1] | 0 | 0.11 [0 to 1] | 0 | Inf |
| *Cx.* (*Cux.*) *hutchinsoni* | 9 | 0.02 [0 to 1] | 0.01 [0 to 1] | 0.14 [0 to 1] | 0.7 | 10 |
| *Cx.* (*Cux.*) *mimulus*/*murelli* | 1 | 0.01 [0 to 1] | 0 | 0 | 0 | NA |
| *Cx.* (*Cux.*) *perplexus*/*whitei* | 2 | 0.01 [0 to 1] | 0 | 0 | 0 | NA |
| *Cx.* (*Cux.*) *quinquefasciatus* | 2 | NA | NA | NA | NA | NA |
| *Cx.* (*Cux.*) *sitiens* | 19 | 0 | 0.01 [0 to 1] | 0.64 [0 to 4] | Inf | 90 |
| *Cx.* (*Cux.*) *tritaeniorhynchus* | 141 | 0.01 [0 to 1] | 0.06 [0 to 3] | 4.64 [0 to 18] | 4.5 | 72.2 |
| *Cx.* (*Cux.*) *vishnui* | 154 | 0.08 [0 to 2] | 0.11 [0 to 2] | 4.43 [0 to 15] | 1.5 | 38.8 |
| *Cx.* (*Cux.*) *whitmorei* | 145 | 0.04 [0 to 1] | 0.17 [0 to 3] | 4.11 [0 to 13] | 4 | 24 |
| *Cx.* (*Cui.*) *baily* | 1 | 0 | 0.01 [0 to 1] | 0 | Inf | 0 |
| *Cx.* (*Cui.*) *nigropunctatus* | 1 | NA | NA | NA | NA | NA |
| *Cx.* (*Cui.*) *brevipalpis*/*phangngae* | 21 | 0.07 [0 to 2] | 0.04 [0 to 1] | 0.18 [0 to 2] | 0.5 | 5 |
| *Cx.* (*Eum.*) *kiriensis* | 2 | 0.01 [0 to 1] | 0 | 0 | 0 | NA |
| *Cx.* (*Eum.*) *malayi* | 1 | 0.01 [0 to 1] | 0 | 0 | 0 | NA |
| *Cx.* (*Lop.*) *mammilifer* | 24 | 0.06 [0 to 2] | 0.07 [0 to 1] | 0.11 [0 to 1] | 1.2 | 1.5 |
| *Cx.* (*Lop.*) *reidi* | 1 | 0 | 0 | 0.04 [0 to 1] | NA | Inf |
| *Cx.* (*Ocu.*) *bitaeniorhynchus* | 49 | 0.13 [0 to 5] | 0.11 [0 to 2] | 0.54 [0 to 2] | 0.8 | 5 |
| *Cx.* (*Ocu.*) *infula* | 1 | 0 | 0.01 [0 to 1] | 0 | Inf | 0 |
| *Cx.* (*Ocu.*) *pseudosinensis* | 2 | 0 | 0 | 0.04 [0 to 1] | NA | Inf |
| *Cx.* (*Ocu.*) *sinensis* | 5022 | 8.16 [0 to 45] | 12.42 [0 to 98] | 55.46 [1 to 416] | 1.5 | 4.5 |
| *Dn. albotaeniata* | 10 | 0.03 [0 to 1] | 0.01 [0 to 1] | 0.04 [0 to 1] | 0.5 | 2.5 |
| *Do. albolateralis* | 2 | 0 | 0 | 0.04 [0 to 1] | NA | Inf |
| *Do. ganapathi* | 84 | 0.31 [0 to 4] | 0.23 [0 to 3] | 0.11 [0 to 1] | 0.7 | 0.5 |
| *Do. harinasutai* | 223 | 0.83 [0 to 7] | 0.59 [0 to 9] | 0.64 [0 to 4] | 0.7 | 1.1 |
| *Do. mikrokopion* | 303 | 1.02 [0 to 12] | 0.89 [0 to 13] | 1.04 [0 to 6] | 0.9 | 1.2 |
| *Do. niveoides* | 1031 | 3.39 [0 to 44] | 3.19 [0 to 47] | 3.36 [0 to 9] | 0.9 | 1.1 |
| *Do. novonivea* | 206 | 0.78 [0 to 7] | 0.55 [0 to 5] | 0.36 [0 to 4] | 0.7 | 0.6 |
| *Do. pexa*/*vana* | 21 | 0.06 [0 to 2] | 0.07 [0 to 4] | 0.04 [0 to 1] | 1.2 | 0.5 |
| *Do.* species 1 | 65 | 0.16 [0 to 2] | 0.22 [0 to 4] | 0.18 [0 to 1] | 1.4 | 0.8 |
| *Fl. flavipennis* | 1 | 0 | 0 | 0.04 [0 to 1] | NA | Inf |
| *Fl. poicilia* | 398 | 1.07 [0 to 11] | 0.81 [0 to 5] | 4.18 [0 to 16] | 0.8 | 5.1 |
| *Fr. vittatus* | 2 | 0 | 0 | 0.04 [0 to 1] | NA | Inf |
| *Hl. chrysolineata* | 7 | 0.01 [0 to 1] | 0.01 [0 to 1] | 0.07 [0 to 1] | 0.5 | 10 |
| *Hl. formosensis* | 1 | 0 | 0.01 [0 to 1] | 0 | Inf | 0 |
| *Hl. harveyi* | 3 | 0.01 [0 to 1] | 0.01 [0 to 1] | 0 | 1 | 0 |
| *Hz.* (*Hez.*) *aureochaeta* | 48 | 0.12 [0 to 3] | 0.18 [0 to 5] | 0 | 1.5 | 0 |
| *Hz.* (*Hez.*) *chengi* | 38 | 0.09 [0 to 3] | 0.11 [0 to 3] | 0.04 [0 to 1] | 1.2 | 0.3 |
| *Hz.* (*Hez.*) *macdonaldi* | 3 | 0 | 0.01 [0 to 1] | 0 | Inf | 0 |
| *Hz.* (*Hez.*) *mattinglyi* | 27 | 0.11 [0 to 4] | 0.07 [0 to 2] | 0.04 [0 to 1] | 0.7 | 0.5 |
| *Hz.* (*Hez.*) *reidi* | 256 | 0.62 [0 to 11] | 0.79 [0 to 11] | 0.5 [0 to 4] | 1.3 | 0.6 |
| *Hz.* (*Hez.*) *scintillans* | 203 | 0.46 [0 to 9] | 0.74 [0 to 10] | 0.14 [0 to 2] | 1.6 | 0.2 |
| *Hz.* (*Mat.*) *achaetae* | 5 | 0.01 [0 to 1] | 0.01 [0 to 1] | 0 | 1 | 0 |
| *Hz.* (*Mat.*) *catesi* | 19 | 0.07 [0 to 4] | 0.05 [0 to 2] | 0 | 0.7 | 0 |
| *Ma.* (*Mnd.*) *uniformis* | 23 | 0.04 [0 to 1] | 0.02 [0 to 1] | 0.5 [0 to 7] | 0.5 | 23.3 |
| *Ma.* (*Mnd.*) *annulata* | 71 | 0.1 [0 to 3] | 0.14 [0 to 4] | 1.25 [0 to 9] | 1.4 | 8.8 |
| *Ma.* (*Mnd.*) *dives* | 6 | 0.02 [0 to 3] | 0 | 0.11 [0 to 3] | 0 | Inf |
| *Mi.* (*Ing.*) *fusca* | 4 | 0 | 0.01 [0 to 1] | 0.07 [0 to 1] | Inf | 10 |
| *Pe.* (*Agl.*) *whartoni* | 2 | 0 | 0.01 [0 to 1] | 0 | Inf | 0 |
| *Pe.* (*Pet.*) *iyengari* | 20 | 0.04 [0 to 1] | 0.09 [0 to 1] | 0 | 2.2 | 0 |
| *Pe.* (*Pet.*) *scanloni* | 6 | 0.02 [0 to 2] | 0.02 [0 to 1] | 0 | 1 | 0 |
| *Ph. prominens* | 1 | 0 | 0.01 [0 to 1] | 0 | Inf | 0 |
| *Ae.* (*Stg.*) *annandalei* | 14 | 0.04 [0 to 1] | 0.05 [0 to 3] | 0.04 [0 to 1] | 1.4 | 0.7 |
| *Ae.* (*Stg.*) *craggi* | 3 | 0.01 [0 to 1] | 0.01 [0 to 1] | 0 | 0.5 | 0 |
| *Ae.* (*Stg.*) *malikuli*/*perplexus* | 21 | 0.06 [0 to 1] | 0.05 [0 to 1] | 0.04 [0 to 1] | 0.8 | 0.7 |
| *Ae.* (*Stg.*) *desmotes* | 25 | 0.07 [0 to 2] | 0.09 [0 to 2] | 0.11 [0 to 2] | 1.2 | 1.2 |
| *Ae. (Stg.) albopictus* | 213 | 0.49 [0 to 5] | 0.88 [0 to 10] | 0.39 [0 to 3] | 1.8 | 0.4 |
| *Ae. (Stg.) gardnerii imitator* | 5 | 0.01 [0 to 1] | 0.02 [0 to 1] | 0 | 1.5 | 0 |
| *Ae. (Stg.) pseudoalbopictus* | 107 | 0.24 [0 to 5] | 0.47 [0 to 6] | 0.14 [0 to 2] | 1.9 | 0.3 |

*Abbreviations*: Inf, infinite; NA, not applicable.
