# Supplementary figures and images for: Assessment of mosquito species communities biting humans and their livestock in the forest hills of Karen state, Myanmar: a cross-sectional survey in six villages
Source: Parasit Vectors. 2025 Dec 29;19:58. doi: 10.1186/s13071-025-07217-9 (PMC12860035; doi:10.1186/s13071-025-07217-9)

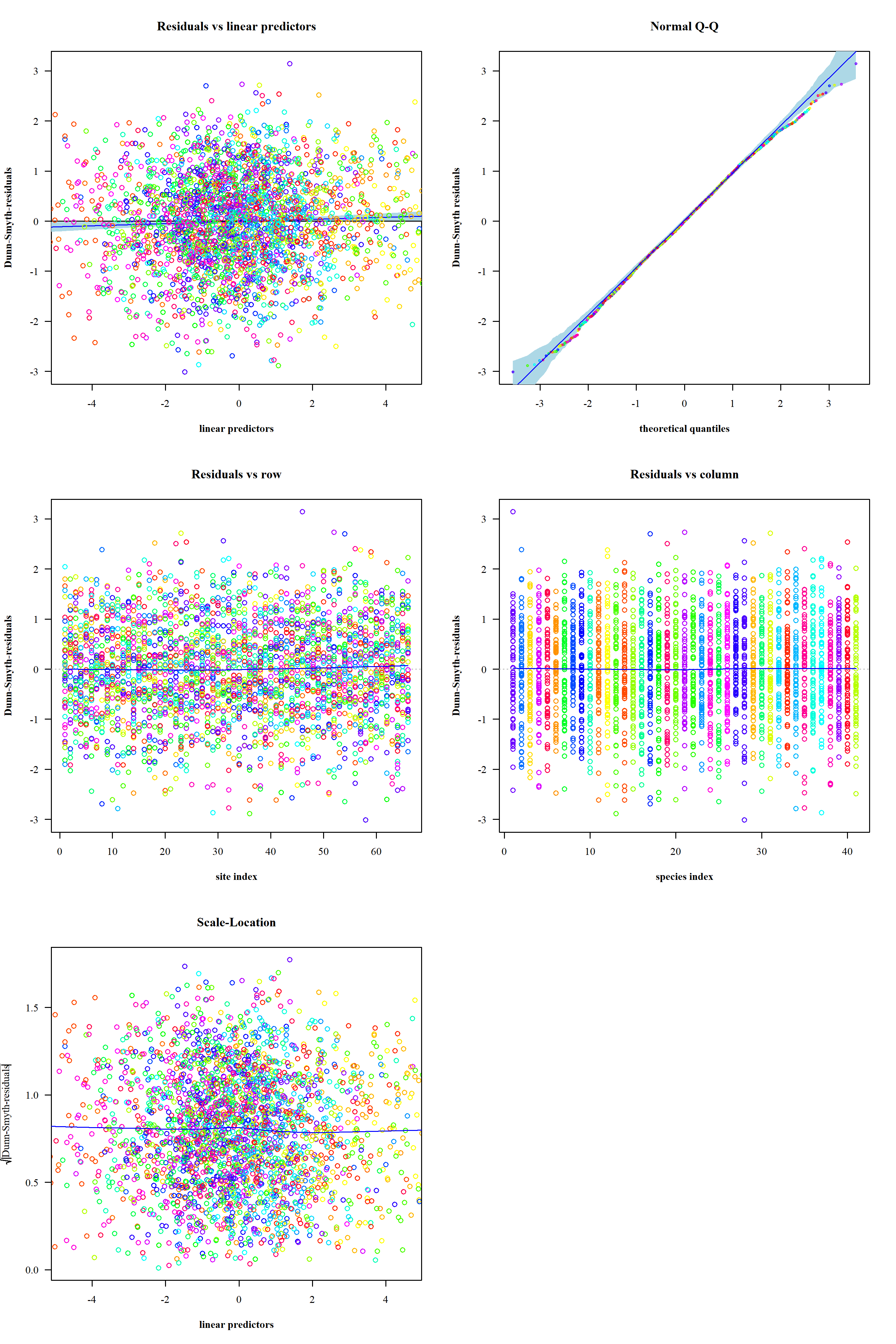

Supplement: Supplementary file 4 — Additional file 4: Figure S1. Diagnostic plots for the negative binomial generalized linear latent variable model without environmental variables. [file 13071_2025_7217_MOESM4_ESM.png]

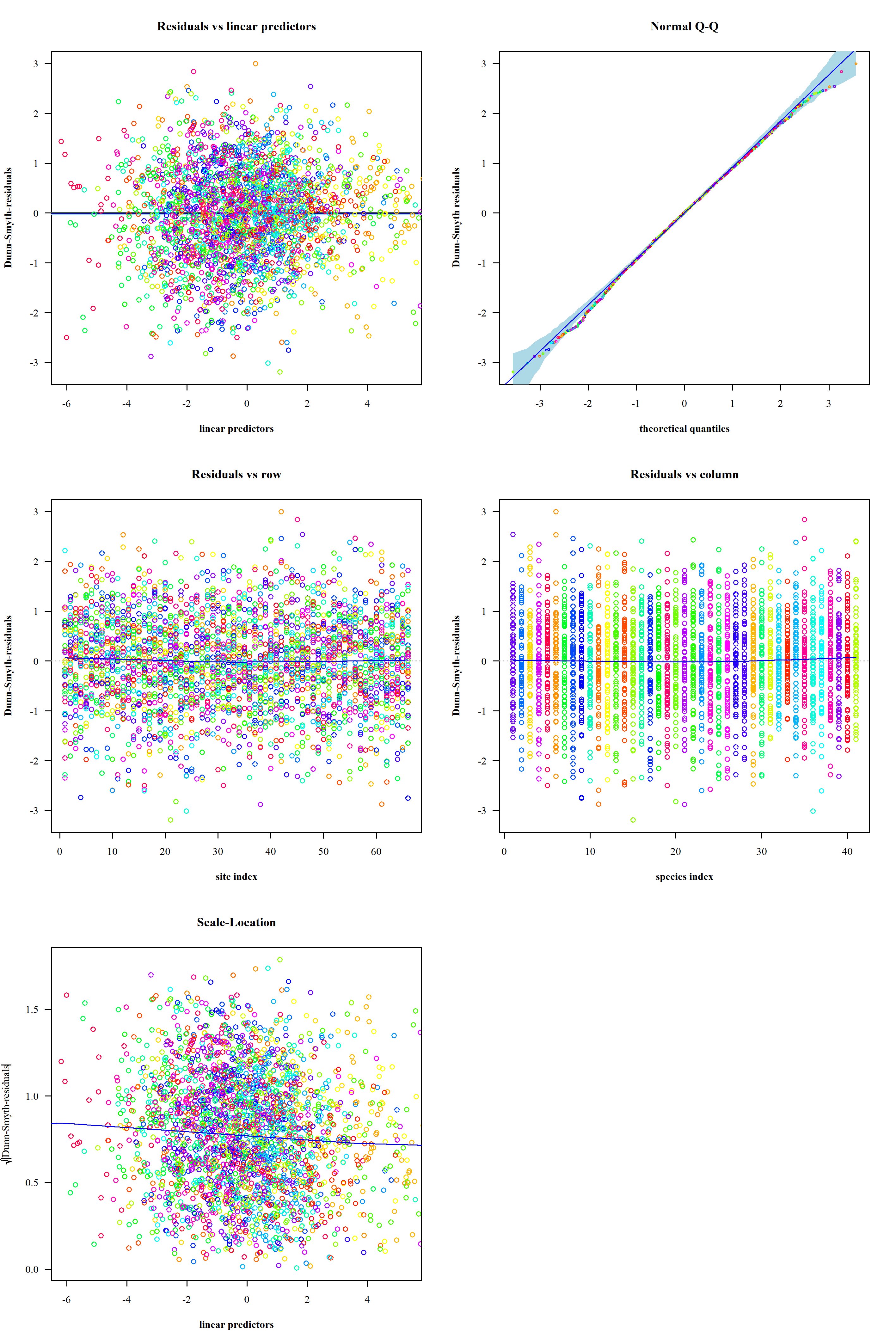

Supplement: Supplementary file 5 — Additional file 5: Figure S2. Diagnostic plots for the negative binomial generalized linear latent variable model with environmental variables. [file 13071_2025_7217_MOESM5_ESM.png]

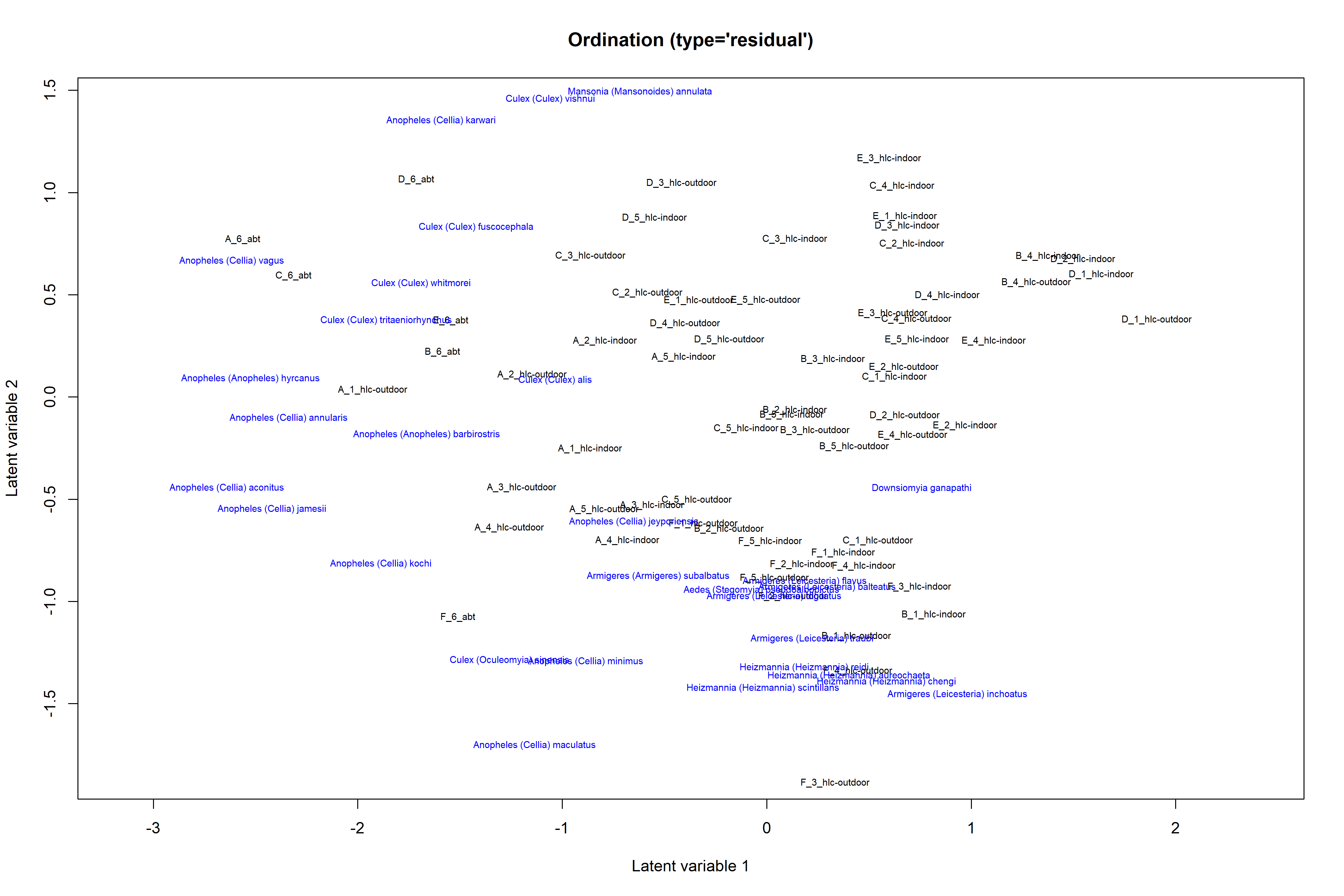

Supplement: Supplementary file 6 — Additional file 6: Figure S3. Ordination plot with 30 indicator species based on the negative binomial generalized linear latent variable model fitted to the mosquito data without environmental variables. [file 13071_2025_7217_MOESM6_ESM.png]

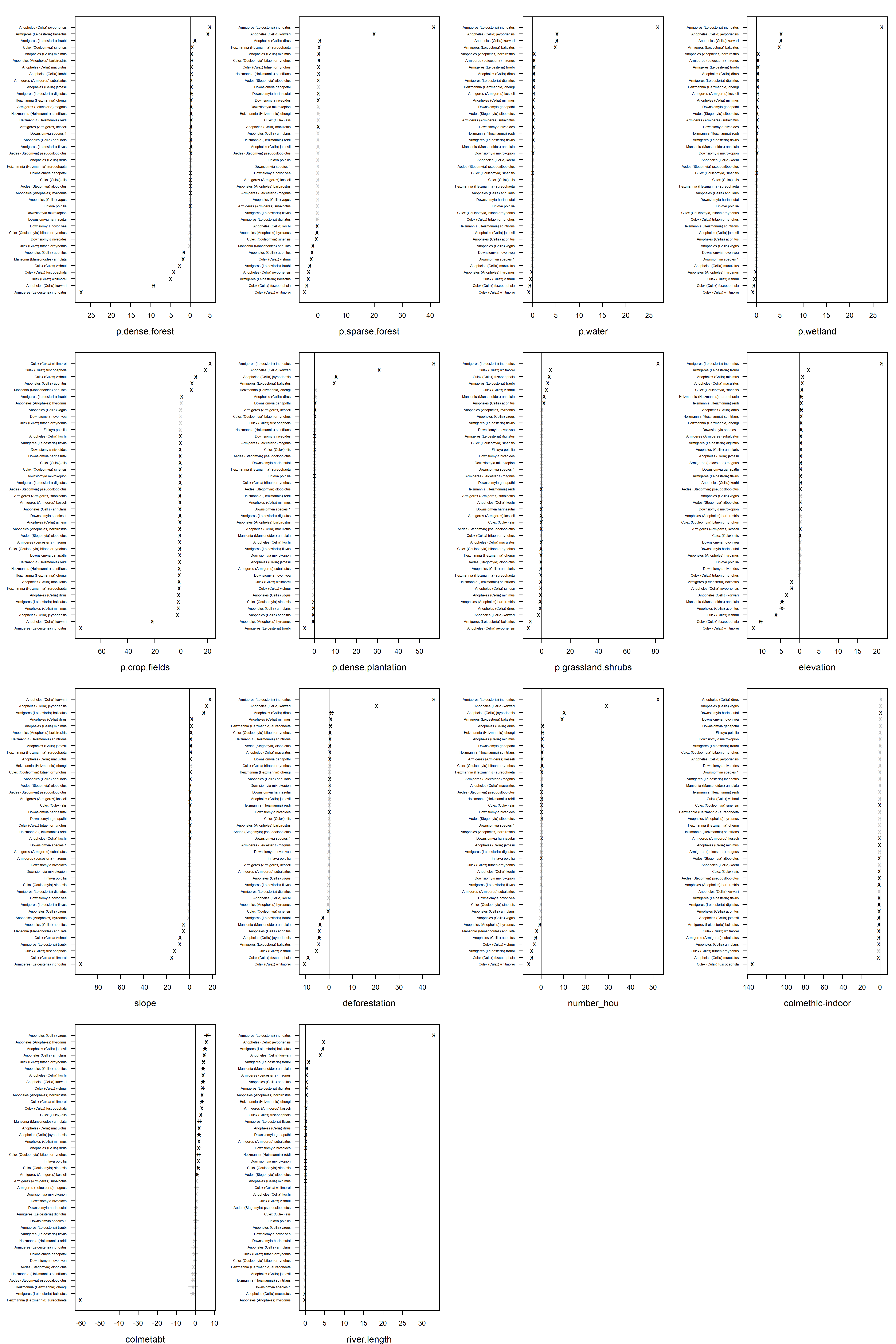

Supplement: Supplementary file 7 — Additional file 7: Figure S4. Plots of the point estimates (ticks) for coefficients of the environmental variables and their 95% confidence intervals (lines) for the negative binomial generalized linear latent variable model, with those coloured in grey (black) denoting intervals (not) containing zero. [file 13071_2025_7217_MOESM7_ESM.png]

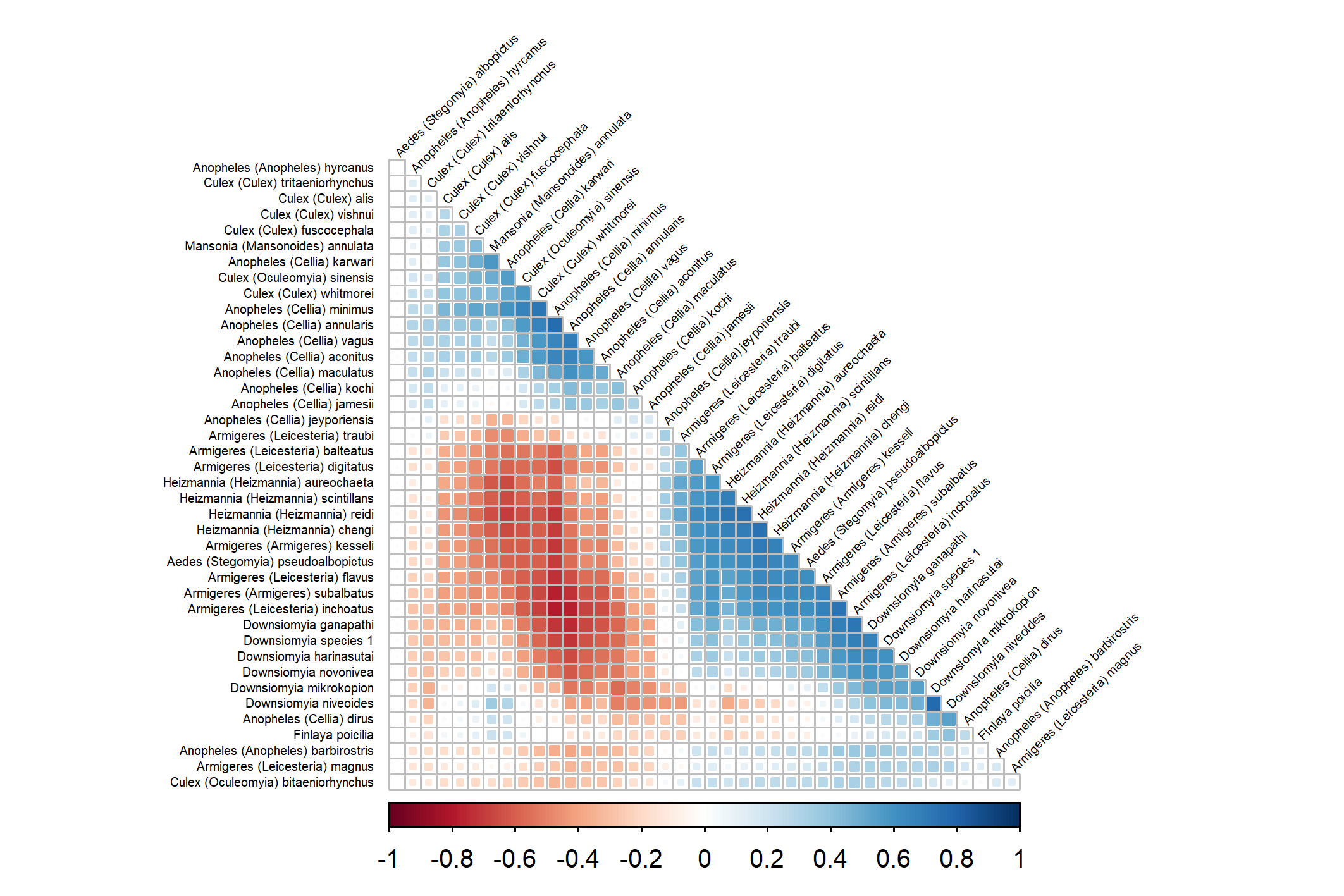

Supplement: Supplementary file 8 — Additional file 8: Figure S5. Residual correlation matrix based on latent factor loadings for the negative binomial generalized linear latent variable model with environmental covariates. [file 13071_2025_7217_MOESM8_ESM.png]
